# Advances in Atherosclerosis Theranostics Harnessing Iron Oxide‐Based Nanoparticles

**DOI:** 10.1002/advs.202308298

**Published:** 2024-02-17

**Authors:** Shi Wang, Hongliang He, Yu Mao, Yu Zhang, Ning Gu

**Affiliations:** ^1^ State Key Laboratory of Digital Medical Engineering Jiangsu Key Laboratory for Biomaterials and Devices School of Biological Sciences & Medical Engineering Southeast University Nanjing 210009 P. R. China; ^2^ School of Medicine Nanjing University Nanjing 210093 P. R. China

**Keywords:** atherosclerosis, iron oxide nanoparticles, nanomedicines, theranostics

## Abstract

Atherosclerosis, a multifaceted chronic inflammatory disease, has a profound impact on cardiovascular health. However, the critical limitations of atherosclerosis management include the delayed detection of advanced stages, the intricate assessment of plaque stability, and the absence of efficacious therapeutic strategies. Nanotheranostic based on nanotechnology offers a novel paradigm for addressing these challenges by amalgamating advanced imaging capabilities with targeted therapeutic interventions. Meanwhile, iron oxide nanoparticles have emerged as compelling candidates for theranostic applications in atherosclerosis due to their magnetic resonance imaging capability and biosafety. This review delineates the current state and prospects of iron oxide nanoparticle‐based nanotheranostics in the realm of atherosclerosis, including pivotal aspects of atherosclerosis development, the pertinent targeting strategies involved in disease pathogenesis, and the diagnostic and therapeutic roles of iron oxide nanoparticles. Furthermore, this review provides a comprehensive overview of theranostic nanomedicine approaches employing iron oxide nanoparticles, encompassing chemical therapy, physical stimulation therapy, and biological therapy. Finally, this review proposes and discusses the challenges and prospects associated with translating these innovative strategies into clinically viable anti‐atherosclerosis interventions. In conclusion, this review offers new insights into the future of atherosclerosis theranostic, showcasing the remarkable potential of iron oxide‐based nanoparticles as versatile tools in the battle against atherosclerosis.

## Graphical abstract

This review mainly focuses on the advances and prospects in the iron oxide nanoparticles‐based atherosclerosis theranostics, which may simultaneously possess targeting, diagnostic, and therapeutic functions. A comprehensive and detailed summary of related researches are provided according to three therapeutic strategies, encompassing the chemical therapy, physical stimulation therapy, and biologic therapy.

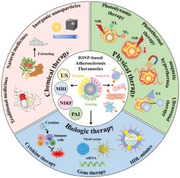

## 1 Introduction

Cardiovascular disease stands as the predominant cause of morbidity and mortality on a global scale, with its etiology closely intertwined with atherosclerosis (AS).^[^ ^1^ ^]^ Atherosclerosis is recognized as a chronic and progressive inflammatory ailment marked by the accumulation of lipids, deposition of fibrous elements, and calcification within the inner linings of arterial vessels.^[^ ^1b^ ^]^ Serving as the primary pathological foundation for coronary artery diseases, the development of atherosclerotic lesions significantly contributes to plaque rupture, ensuing thrombosis, and consequently, the onset of critical cardiovascular events, including myocardial infarction, acute coronary syndromes, sudden cardiac death, stroke, and other severe cardiovascular complications.^[^ ^2^ ^]^ As a result, early diagnosis and effective treatment of atherosclerosis assume paramount importance in retarding disease progression and averting the emergence of life‐threatening cardiovascular conditions.

At present, clinical strategies for the diagnosis and management of atherosclerosis face numerous limitations and challenges. From a diagnostic perspective, current clinical screening techniques for atherosclerosis primarily encompass methods like transesophageal echocardiography, intravascular ultrasound, computed tomography angiography, and carotid magnetic resonance imaging (MRI).^[^ ^3^ ^]^ However, these traditional approaches proved less effective in early disease detection, failing to identify atherosclerosis before the manifestation of overt clinical symptoms, and primarily offering anatomical and physiological information.^[^ ^4^ ^]^ On the therapeutic front, the conventional approach for atherosclerosis involves medical treatment. Common medications for atherosclerosis encompass lipid‐lowering drugs, antiplatelet agents, vasodilators, and others. Nevertheless, their clinical utility is hampered by side effects and limited bioavailability, resulting from their lack of specificity in atherosclerosis management.^[^ ^5^ ^]^ Furthermore, late‐stage atherosclerosis, often accompanied by myocardial infarction or thrombus rupture, necessitates surgical interventions like stent‐assisted therapies and coronary artery bypass surgery.^[^ ^6^ ^]^ While these surgical interventions significantly reduce disease‐related mortality, they also entail potential complications such as inflammation and thrombosis.^[^ ^7^ ^]^ Therefore, substantial efforts are being devoted to the development of diagnostic and therapeutic approaches for atherosclerosis that prioritize both safety and efficacy.

The rapid advancement of nanotechnology has ushered in the era of nanomedicine, offering novel tools and avenues for the treatment and diagnosis of diseases. Over the past decade, extensive research has focused on nanomaterials as versatile agents, playing critical roles as imaging contrast agents, therapeutic agents, and nanocarriers to drive significant breakthroughs in disease therapy and diagnosis.^[^ ^8^ ^]^ Notably, theranostic nanoplatforms have garnered widespread recognition for their ability to seamlessly integrate multiple functions, including targeting, imaging, and therapy, within a single nano‐system.^[^ ^9^ ^]^ Among the diverse array of nanomaterials, iron oxide nanoparticles (IONPs) have emerged as intensive subjects of study for constructing theranostic nanoplatforms, attributed to their exceptional magnetic properties, size controllability, adaptable size and shape, and ease of surface modification.^[^ ^10^ ^]^ Superparamagnetic iron oxide nanoparticles (SPIONs) are broadly utilized in MRI, hyperthermia, targeted drug delivery, and other fields.^[^ ^11^ ^]^ Importantly, SPIONs have been approved by the Food and Drug Administration (FDA) for biomedical applications, attesting to their biological safety.^[^ ^12^ ^]^ In the context of combating atherosclerosis, iron oxide‐based nanoparticles primarily focus on two key aspects. First, they enhance the sensitivity of MRI for early atherosclerosis diagnosis and the differentiation of various stages of atherosclerotic plaques by targeting diverse atherosclerosis‐related molecules or cells. Second, they function as efficient drug carriers, facilitating the delivery of therapeutic agents and thereby enabling the development of innovative treatment modalities such as ultrasound therapy, photothermal therapy (PTT), photodynamic therapy (PDT), and combination therapy.^[^ ^8^, ^13^ ^]^

**Figure 1:**
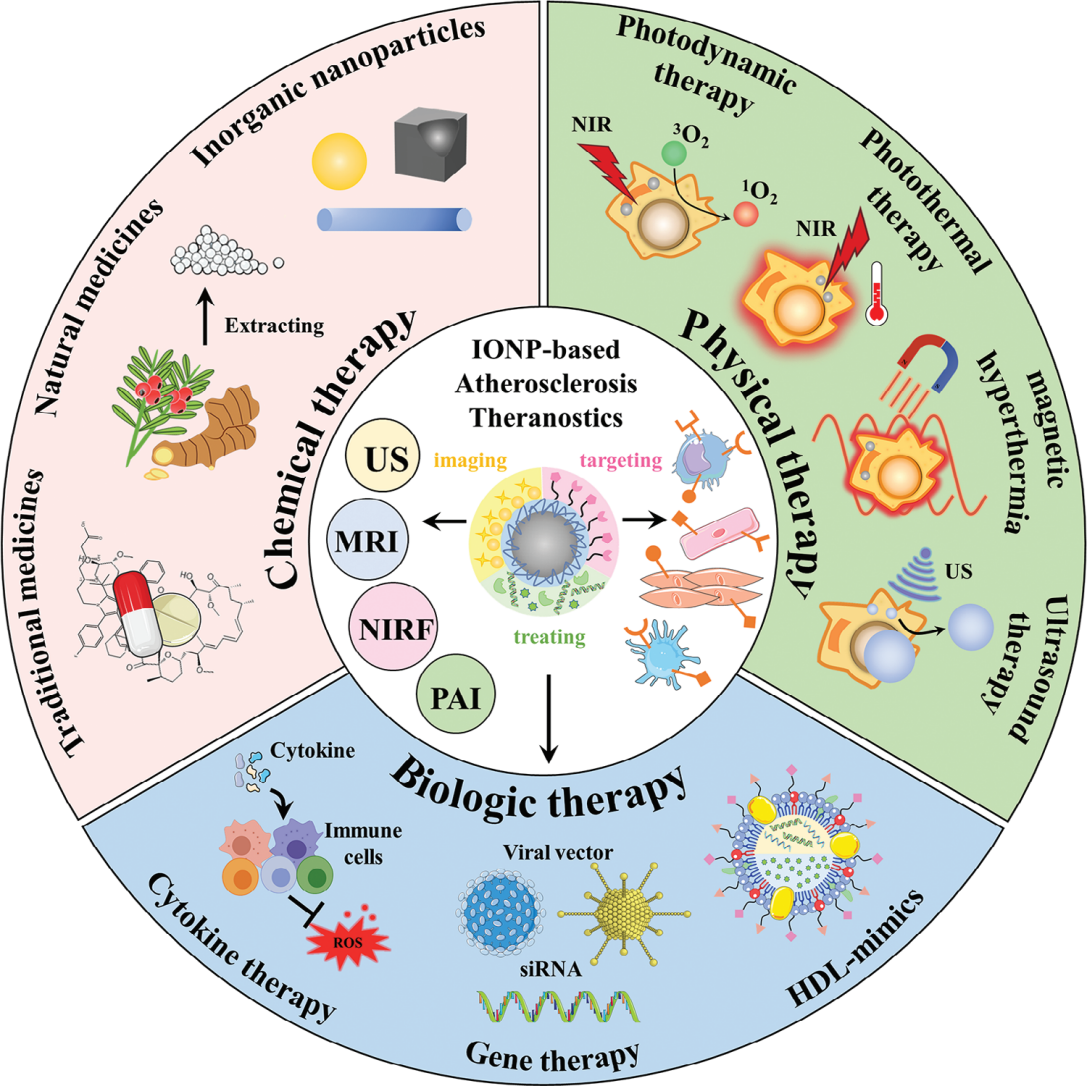
Illustrative overview of therapeutic strategies in IONP‐based theranostics for atherosclerosis.

In this comprehensive review, we begin by providing a concise overview of the pathological transformations pivotal to the progression of atherosclerosis and then summarize primary active targeting strategies of atherosclerosis‐related to the application of IONPs. Subsequently, we introduce and analyze the realm of iron oxide nanoparticles and their indispensable role in atherosclerosis. The focus of this review then shifts to a systematic exploration of IONP‐based theranostic nanomedicine for atherosclerosis, categorized according to therapeutic strategies, as depicted in **Figure** **1**. These strategies include chemical therapy, physical stimulation therapy, and biological therapy. Finally, this review not only concludes the potential prospects but also discusses the inherent challenges in the application of IONP‐based nanomedicine for atherosclerosis theranostics, guiding future research directions.

## 2 Overview of Atherosclerosis and Iron Oxide Nanoparticles

### 2.1 Overview: Pathophysiology of Atherosclerotic Development

**Figure 2:**
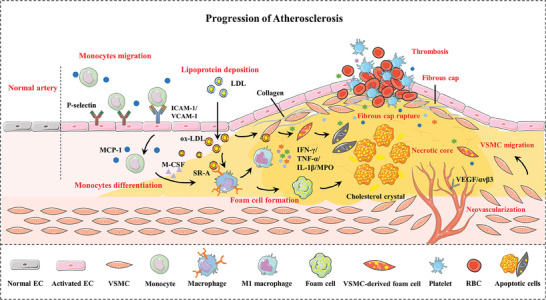
Schematic illustration of the development and pathological characteristics of atherosclerosis. ECs, endothelial cells; ox‐LDL, oxidized low‐density lipoprotein; ICAM‐1, intercellular adhesion molecule‐1; VCAM‐1, vascular cell adhesion molecule‐1; MCP‐1, monocyte chemotactic protein‐1; M‐CSF, macrophage colony‐stimulating factors; VSMCs, vascular smooth muscle cells; IFN‐γ, interferon‐γ; IL‐1β, interleukin‐1β; TNF‐α, tumor necrosis factor‐α; MPO, myeloperoxidase; VEGF, vascular endothelial growth factor.

The development of atherosclerosis is a multifaceted and continuous process characterized by a series of intricate physiological events. These events include endothelial dysfunction, lipoprotein deposition, monocyte migration and differentiation, inflammatory responses, angiogenesis, plaque formation, and ultimately, the potential for plaque rupture, leading to thrombosis or stenosis.^[^ ^14^ ^]^ Typically, atherosclerosis tends to manifest in arterial branches or curvatures characterized by turbulent blood flow and low shear stress.^[^ ^15^ ^]^ Currently, there is a profound understanding of the pathological progression of atherosclerosis, as shown in **Figure** **2**, which can be categorized into three distinct stages: early, intermediate, and advanced.

The initial stage of atherosclerosis is characterized by endothelial dysfunction as a primary trigger, which leads to increased permeability to lipoproteins. Subsequently, circulating low‐density lipoprotein (LDL) particles, breach the endothelial layer and accumulate in the subendothelial matrix, transforming into oxidized LDL (ox‐LDL).^[^ ^16^ ^]^ Meanwhile, endothelial cells (ECs) become stimulated and activated, upregulating the expression of inflammatory cytokines, leucocyte adhesion molecules, chemokines, etc., thereby facilitating monocyte recruitment. Among these molecules, P‐selectin, intercellular adhesion molecule‐1 (ICAM‐1), vascular cell adhesion molecule‐1 (VCAM‐1), and monocyte chemotactic protein‐1 (MCP‐1) play pivotal roles in the transendothelial migration of monocyte.^[^ ^17^ ^]^ Upon entering the intima space, monocytes are regulated by macrophage colony‐stimulating factors(M‐CSF) and undergo differentiation into macrophages. These macrophages, along with vascular smooth muscle cells (VSMCs), engage in the phagocytosis of ox‐LDL, ultimately resulting in foam cell formation. Foam cells constitute a significant portion of the early fatty streak and serve as a hallmark of early atherosclerosis.^[^ ^1^, ^18^ ^]^

The emergence of the necrotic core and fibrous cap signifies the second stage of atherosclerosis. During this phase, in response to damaging stimuli, VSMCs transition from a quiescent contractile phenotype to a synthetic phenotype, driving the proliferation and migration of VSMCs into the intima under the facilitation of growth factors like vascular endothelial growth factor (VEGF).^[^ ^19^ ^]^ Furthermore, VSMCs in the synthetic state secrete extracellular matrix components such as interstitial collagen, elastin, and proteoglycans. These elements collaboratively form a fibrous cap that envelopes the atherosclerotic plaque, shielding it from rupture.^[^ ^20^ ^]^ Conversely, the necrotic core concealed by the fibrous cap consists of cholesterol, cellular debris, and diminished supportive collagen.^[^ ^18^, ^21^ ^]^ As the accumulation, apoptosis, and necrosis of foam cells, the necrotic core gradually expands, accordingly rendering the plaque increasingly vulnerable.^[^ ^22^ ^]^

In the advanced stage of atherosclerosis, vulnerable plaques exhibit distinct characteristics, including a large necrotic core, a thin fibrous cap, and continuous exposure to a pro‐atherogenic milieu.^[^ ^23^ ^]^ These plaques are particularly prone to rupture due to neovascularization and constant erosion from hemodynamic forces, causing the necrotic core exposed to the bloodstream.^[^ ^13^, ^23^ ^]^ Afterward, this exposure triggers a coagulation process, activating platelets to recruit and aggregate at the rupture site, eventually leading to thrombosis.^[^ ^24^ ^]^ More severely, thrombosis can give rise to complications such as ischemic cardiopathies, myocardial infarction, or stroke due to vessel obstruction. In unfortunate instances, the thrombus may detach from the arterial wall and become lodged in a distal vessel, posing a significant threat to overall blood circulation and blood supply to various organs.^[^ ^1^, ^23^, ^25^ ^]^

### 2.2 Targets of Atherosclerosis Related to the Application of Iron Oxide Nanoparticles

**Figure 3:**
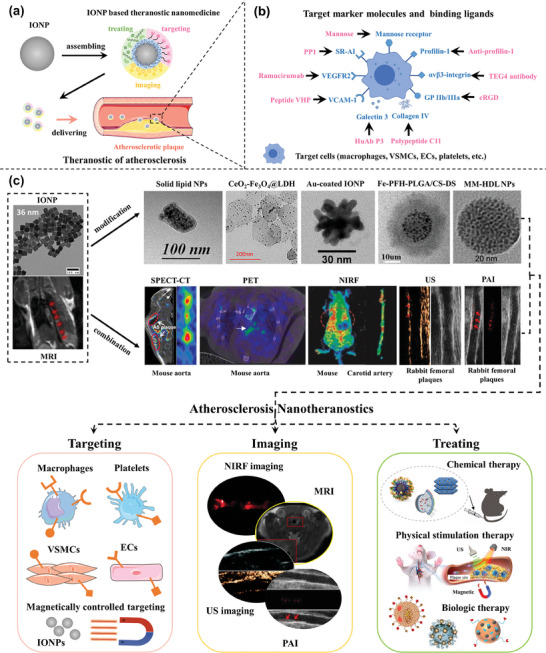
a) Schematic representation of theranostic nanomedicine for atherosclerosis. b) Target marker molecules and binding ligands for atherosclerosis Theranostics. c) Diverse IONP‐based theranostic nanoplatforms for atherosclerosis targeting, imaging, and treatment. Representative image of IONPs. Reproduced with permission.^[^ ^60^ ^]^ Copyright 2018, American Chemical Society. Image of MRI (up) and MM‐HDL NPs (TEM and figure in biologic therapy). Reproduced with permission.^[^ ^61^ ^]^ Copyright 2020, American Chemical Society. Image of solid lipid nanoparticles (TEM and figure in chemical therapy). Reproduced with permission.^[^ ^62^ ^]^ Copyright 2016, American Chemical Society. Image of CeO_2_‐ Fe_3_O_4_@LDH nanocomposites (TEM and figure in chemical therapy). Reproduced with permission.^[^ ^63^ ^]^ Copyright 2019, American Chemical Society. Image of Au‐coated IONP. Reproduced with permission.^[^ ^64^ ^]^ Copyright 2009, American Chemical Society. Image of Fe‐PFH‐PLGA/CS‐DS NPs. Reproduced with permission.^[^ ^65^ ^]^ Copyright 2019, American Chemical Society. Image of SPECT‐CT. Reproduced with permission.^[^ ^66^ ^]^ Copyright 2018, American Chemical Society. Image of PET. Reproduced under terms of the CC‐BY license.^[^ ^67^ ^]^ Copyright 2021, The Authors, Published by American Chemical Society. Image of NIRF (up) and MRI (down). Reproduced under terms of the CC‐BY license.^[^ ^68^ ^]^ Copyright 2022, The Authors, Published by MDPI, Basel, Switzerland. Image of NIRF (down) and nanoparticle figure in chemical therapy. Reproduced under terms of the CC‐BY license.^[^ ^38^ ^]^ Copyright 2019, The Authors, Published by Informa UK Limited, trading as Taylor & Francis Group. Image of US and PAI. Reproduced under terms of the CC‐BY license.^[^ ^69^ ^]^ Copyright 2021, Published by Wiley‐VCH. Image of physical stimulation therapy. Reproduced with permission.^[^ ^70^ ^]^ Copyright 2022, American Chemical Society. Image of nanoparticles in biologic therapy. (middle) Reproduced with permission.^[^ ^71^ ^]^ Copyright 2016, Elsevier B.V. (right) Reproduced with permission.^[^ ^72^ ^]^ Copyright 2020, Elsevier Ltd.

The development of atherosclerosis reveals that ECs, monocytes, macrophages, VSMCs, platelets, and even cellular molecules actively engage in numerous critical processes and occupy considerable roles. Consequently, they are commonly employed as target cells or molecules for imaging diagnosis and active targeting therapy in the various stages of atherosclerosis.^[^ ^26^ ^]^ This section superficially summarizes the main active targeting strategies of atherosclerosis‐related to the application of Iron Oxide Nanoparticles (**Figure** **3**b).

#### 2.2.1 Targeting ECs

ECs emerge as primary targets due to their crucial role in the early stages of atherosclerosis. In targeting ECs, cell adhesion molecules like VCAM‐1, ICAM‐1, and P‐selectins, which are overexpressed on the ECs' surface, have proven to be both commonly utilized and effective molecular targets.^[^ ^27^ ^]^ VCAM‐1, an adhesion molecule prominently expressed on activated ECs, plays a critical role in recruiting leukocytes to the vascular endothelium. It exhibits a high affinity and interacts specifically with leukocytes, primarily through its recognition and binding to the α4β1 integrin glycoprotein abundantly expressed on the surfaces of leukocytes and macrophages. As such, VCAM‐1 has been employed as a biomarker for identifying abnormal ECs. The anti‐VCAM‐1 antibody conjugated to IONPs has been proven effective in identifying atherosclerotic plaques in the ApoE^−/−^ mouse model by MRI.^[^ ^28^ ^]^ Previous research has also demonstrated that IONPs coated with leukocyte or macrophage membranes, or functionalized with targeted peptides (such as the VHPKQHR peptide), which shares homology with α4β1 integrin, can effectively target ECs within atherosclerotic plaques.^[^ ^29^ ^]^

#### 2.2.2 Targeting Macrophages

During the mid‐to‐advanced stages of atherosclerosis, a complex interplay of cellular physiological activities and related biomolecules takes place within the plaque site. Among these, macrophages, being the most abundant and central cells within atherosclerotic plaques, offer a rich array of targets and biomarkers for plaque targeting.^[^ ^16^, ^30^ ^]^ These include scavenger receptors such as scavenger receptor type AI (SR‐AI) and CD36,^[^ ^31^ ^]^ receptors overexpressed on activated macrophages like folate receptors, mannose receptors, and transferrin receptor 1,^[^ ^32^ ^]^ inflammatory components such as CD44 receptors and IL‐6 cytokines,^[^ ^33^ ^]^ as well as biomarkers associated with M1 macrophages such as CD68 receptors and MARCO,^[^ ^34^ ^]^ among others. It's worth noting that specific interactions have been identified in the application of IONPs, such as PP1's affinity for binding with SR‐AI.^[^ ^35^ ^]^ In addition, IONPs modified with hyaluronic acid can target macrophages due to their interaction with the macrophage surface receptor CD44.^[^ ^36^ ^]^ Inflammatory biomarkers further include myeloperoxidase (MPO), a heme peroxidase whose oxidative products contribute to the development of vulnerable plaques and can be targeted by 5‐hydroxytryptamine. The study of a novel multimodal imaging agent based on SPIONs, targeting active MPO, has revealed that active MPO‐targeted nanoparticles might function as an approach for detecting vulnerable atherosclerotic plaques.^[^ ^37^ ^]^

#### 2.2.3 Targeting VSMCs

Expanding beyond macrophages, the dynamic changes in VSMCs, including their phenotype transition, proliferation, and migration characteristics, further broaden the scope and possibilities for active targeting in atherosclerosis by focusing on molecules related to VSMCs. Profilin‐1, a multifunctional actin‐binding protein known for its role in inducing VSMC migration and participating in vascular remodeling, has demonstrated specific targeting potential for VSMCs through conjugation with a profilin‐1 antibody (PFN1).^[^ ^38^ ^]^ Moreover, various integrin receptors, including ανβ1 and ανβ3, are expressed on vascular cells. The researchers have applied specifically targeted peptides to match the functional domains of ανβ3, such as the RGD and SVVYGLR sequences, to target atherosclerosis.^[^ ^39^ ^]^

#### 2.2.4 Targeting Platelets

Platelets are involved in the process of atherosclerosis lesions through the interaction with lipids, leukocytes, ECs, and VSMCs. Especially, they are the key mediator for plaque rupture and erosion, thrombosis, and plaque healing.^[^ ^40^ ^]^ Platelet receptors are potential targets for atherosclerosis, including integrin glycoprotein (GP) IIb/IIIa, GPVI, thrombin receptors, etc.^[^ ^41^ ^]^ GP IIb/IIIa highly expressed on the surface of activated platelets can bind with fibrinogen, and further promote platelet aggregation. The cRGD peptide is highly adhesive to the GP IIb/IIIa complex and has emerged as the targeting ligand coupling with IONPs to target platelets at the plaque site in some reported studies.^[^ ^35^ ^]^ Additionally, the platelet‐specific collagen receptor GPVI, mediating platelet activation, adhesion, and aggregation, has been studied as a promising antiplatelet target for atherosclerosis.^[^ ^42^ ^]^

#### 2.2.5 Other targets

Beyond the cell‐centered targeting strategies mentioned earlier, researchers have explored and developed additional approaches, such as targeting neovascularization or the extracellular matrix in the application of IONPs.^[^ ^16^, ^43^ ^]^ Numerous biomarkers, including VEGF, ανβ3, and hypoxia‐inducible factors, participate in neovascularization, making them potential targets for plaque visualization and treatment.^[^ ^44^ ^]^ The extracellular matrix, enriched in collagen, serves as a critical component of the fibrous cap, primarily responsible for maintaining its structural integrity. Consequently, several studies centered on IONPs have focused on stabilizing plaques by delivering nanomedicines targeted to type IV collagen.^[^ ^45^ ^]^

### 2.3 Iron Oxide Nanoparticles and Their Application in Atherosclerosis

Iron oxide nanoparticles (IONPs) represent a crucial category of magnetic nanomaterials, including notable varieties such as magnetite (Fe_3_O_4_), hematite (α‐Fe_2_O_3_), maghemite (γ‐Fe_2_O_3_), and mixed ferrites. Due to their superparamagnetic properties, high biocompatibility, and distinctive catalytic attributes, IONPs have emerged as promising diagnostic and therapeutic agents across a wide spectrum of diseases.^[^ ^46^ ^]^

In the realm of medical imaging, IONPs have been extensively investigated as MRI contrast agents. They can modulate signal intensity by altering the relaxation properties of hydrogen protons in surrounding tissues, thereby enhancing tissue differentiation and contrast.^[^ ^47^ ^]^ Traditionally, IONPs with high T_2_ relativity have been employed for T_2_‐weighted MRI, leading to darkened contrasts in regions where they accumulate.^[^ ^48^ ^]^ However, this approach poses limitations, as it may result in false‐positive detections due to potential confusion between lesions and bleeding, calcification, or metal deposition.^[^ ^46^, ^49^ ^]^ Consequently, IONP‐based T1 contrast agents, providing bright contrasts, have been developed to mitigate these limitations.^[^ ^50^ ^]^ Furthermore, there has been substantial research into the realization of simultaneous T2/T1 and bi‐ or tri‐modal imaging, made achievable by either surface conjugation with secondary imaging components for positron emission tomography (PET), single‐photon emission computed tomography (SPECT), computed tomography (CT), near‐infrared fluorescent (NIRF) imaging, photoacoustic imaging (PAI), or ultrasound (US) imaging^[^ ^46^, ^51^ ^]^ or ion doping (e.g., Gd, Mn, Au), as shown in Figure 3c.^[^ ^52^ ^]^

In general, IONPs tend to be phagocytosed by macrophages upon entry into the bloodstream, potentially allowing them to accumulate at inflammatory sites, such as atherosclerotic plaques.^[^ ^53^ ^]^ However, this phagocytosis can lead to rapid nanoparticle metabolism in the body, potentially limiting their effectiveness. Hence, the surface functionalization of IONPs holds immense significance in extending their blood circulation time and improving bioavailability. This can be achieved through various means, including coating them with natural and synthetic polymers like polyethylene glycol (PEG) and dextran,^[^ ^54^ ^]^ or enveloping them in cell membranes such as erythrocyte membranes.^[^ ^55^ ^]^ Surface modification is essential for preventing nanoparticle agglomeration in biological fluids. Additionally, the nanoparticle surface influences protein absorption, resulting in the formation of a protein corona that significantly impacts immune system clearance. Research by Stepien et al. elucidated how the biodistribution and degradation time of IONPs with distinct surface coatings (e.g., PEG or glucose) vary in vivo and in vitro, underscoring the role of surface coating and protein corona absorption in nanoparticle biodegradation and clearance rates.^[^ ^56^ ^]^ In another study, Hu et al. compared the blood circulation time between PEGylated nanoparticles and erythrocyte membrane‐coated nanoparticles, highlighting the superior circulation half‐life of erythrocyte‐mimicking nanoparticles.^[^ ^57^ ^]^ Furthermore, polymer coatings can provide surface reactive sites, such as carboxyl, amino, or thiol groups, for attaching other protective or functional molecules, imparting new characteristics such as pH‐sensitivity or thermo‐sensitivity.^[^ ^58^ ^]^ Furthermore, IONPs can function as potential therapeutic agents, or encapsulate therapeutic agents (e.g., clinical drugs, biopharmaceutical macromolecular drugs, photosensitizers) within materials to construct multifunctional theranostic nanoplatforms for atherosclerosis. For nanoplatforms delivery, the surface of IONPs can be equipped with ligands to facilitate specific interactions and enhance their active targeting to atherosclerosis‐related sites, as discussed in section 2.2.^[^ ^51^, ^59^ ^]^ Alternatively, magnetic fields can guide IONP‐based nanoparticles to their target locations, enabling controlled drug release.^[^ ^51^ ^]^

So far, the research on theranostic applications of IONPs has predominantly centered around cancer, with comparatively less attention directed toward atherosclerosis. Moreover, in the context of atherosclerosis, a greater proportion of articles have concentrated on the diagnostic capabilities of IONP‐based nanoparticles, often delineating diagnostic and therapeutic applications separately. Consequently, IONP‐based theranostic nanoplatforms have somewhat occupied a secondary position in this context. Thus, a comprehensive review of IONP‐based theranostic nanomedicine in the context of atherosclerosis is both timely and pertinent, and the relevant literature is summarized in **Table** **1**.

**Table 1:** Iron Oxide Nanoparticle‐Based Theranostic Nanomedicine with Various Therapeutic Strategies for Atherosclerosis.

Therapeutic strategy	Drug/method	Name of agent	Binding ligand	Target marker	Therapeutic effect	Imaging modality	Ref
Chemical therapy	Rapamycin	RAP@Fe_3_O_4_‐PDA‐CD‐PEG‐PEI‐Profilin‐1‐Cy5.5 nanoparticles	Anti‐profilin‐1	Profilin‐1, vascular smooth muscle cells (VSMCs)	Anti‐inflammatory and stabilized plaque	Magnetic resonance imaging (MRI)/Near‐infrared fluorescent (NIRF)	[38]
	Rapamycin	Rap/Fe_3_O_4_@VHP‐Lipo	VHPKQHR (VHP) peptide	Vascular cell adhesion molecule‐1 (VCAM‐1)	Reduce plaque and lower blood	MRI/Fluorescence bimodal imaging	[68]
	Fumagillin	α_v_β_3_‐targeted SPIONs	Peptidomimetic vitronectin antagonist	α_v_β_3_‐integrin, VSMCs	Inhibit angiogenesis	MRI	[39b]
	Fumagillin; Atorvastatin	α_v_β_3_‐targeted SPIONs	Peptidomimetic α_v_β_3_‐integrin antagonist	α_v_β_3_‐integrin, VSMCs	Inhibit angiogenesis; Lower blood lipid	MR molecular imaging	[39a]
	Dexamethasone (DEXA)	SPION‐DEXA	/	/	Anti‐inflammatory	MRI	[73]
	α‐tocopherol prostacyclin	IONP‐loaded Solid lipid nanoparticles	/	Platelet	Inhibit platelet aggregation	MRI	[62]
	α‐tocopherol	P3‐functionalized NE‐SPIO‐PEG	Fully Human scFv‐Fc Antibody(P3)	Galectin 3, Macrophage	Antioxidation	MRI	[74]
	Protocatechuic acid	MNP‐DEX/PCA	Dextran	Dectin‐1, Macrophage	Anti‐inflammatory	MRI	[54a]
	Curcumin	SDP‐VCAM‐1/Cur/Cy5.5	VCAM‐1 targeted peptide	VCAM‐1	Scavenger ROS, anti‐inflammatory and antioxidant	MRI/Fluorescence imaging	[75]
	Paclitaxel	USPIO + paclitaxel‐loaded polymer‐lipid hybrid theranostic nanoparticles conjugated with C11 (UP‐NP‐C11)	Polypeptide C11	Collagen IV	Inhibit plaque progression and promote plaque stability	MRI	[45c]
	CeO_2_ NPs	Iron oxide–cerium oxide core‐shell nanoparticles	/	/	Scavenger ROS, anti‐inflammatory and antioxidant	MRI	[76]
Chemical therapy	CeO_2_ NPs	CeO_2_·Fe_3_O_4_@LDH	/	/	Scavenger ROS, anti‐inflammatory and antioxidant	MRI	[63]
	Trisodium citrate‐coated cerium oxide	Chitosan Nanococktails containing both Ceria and SIONPs	/	/	Scavenger ROS, anti‐inflammatory and antioxidant	MRI	[77]
Physical stimulation therapy	Photodynamic therapy, chlorin‐based photosensitizer	CLIO‐TPC	Dextran	Dectin‐1, Macrophage	Ablation of macrophages	MRI/NIRF	[78]
	Photodynamic therapy, chlorin‐based photosensitizer	CLIO‐THPC	Dextran	Dectin‐1, Macrophage	Ablation of macrophages	MRI/NIRF	[79]
	Photothermal therapy	Au‐coated IONP nanoclusters	Dextran	Dectin‐1, Macrophage	Ablation of macrophages	MRI/NIRF	[64]
	Photothermal therapy	Ferrite‐encapsulated nanoparticles (PFH@PLGA/MnFe_2_O_4_‐Ram)	Ramucirumab (Ram)	Vascular endothelial growth factor receptor 2 (VEGFR2) on endothelial cells	Inhibit angiogenesis and promote plaque stability	MRI/Photoacoustic (PA)/Ultrasound (US)	[80]
	Magnetic hyperthermia	SPIONs	/	/	Plaque Abrasion	MRI	[81]
	Magnetic hyperthermia; Atorvastatin	pH‐responsive magnetic nanoplatforms (MMNS@AT‐CS‐DS)	Dextran sulfate (DS)	Class A scavenger receptors (SR‐A) on macrophage	Increase protective autophagy and regulate lipid metabolism	MRI	[82]
	Ultrasound therapy, perfluorohexane (PFH)	SR‐A‐targeted phase‐transition nanoparticles (Fe‐PFH‐PLGA/CS‐DS NP)	Dextran sulfate	SR‐A on macrophage	Macrophage apoptosis	MRI/US	[65]
	Ultrasound therapy, perfluorohexane	LIFU‐responsive nanomedicine (FPD@CD)	Dextran sulfate	SR‐A on macrophage	Macrophage apoptosis and stabilize and reduce plaque	MRI/NIRF	[83]
Physical stimulation therapy	Ultrasound therapy, perfluorohexane	Multifunctional pathology‐mapping theranostic nanoplatform (MPmTN)	PP1, cRGD peptide	SR‐A on macrophage, glycoprotein (GP) IIb/IIIa on activated platelets	Macrophage apoptosis and destroy thrombus	MRI/US	[35]
	Sonodynamic therapy, hematoporphyrin monomethyl ether (HMME)	Ultrasound‐Responsive Ferrite‐Encapsulated Nanoparticles (PFP–HMME@PLGA/MnFe_2_O_4_–Ram)	Ramucirumab	VEGFR2 on endothelial cells	Inhibit angiogenesis and promote plaque stability	MRI/PA/US	[69]
Biologic therapy	High‐Density‐Lipoprotein (HDL)‐mimics	High‐Density Lipoprotein‐like Magnetic Nanostructures (HDL‐MNS)	Apolipoprotein A1	Macrophage	Reverse cholesterol transport	MRI	[84]
	HDL‐mimics	Dual‐targeted HDL‐mimicking nanoparticles	Mannose, triphenyl‐phosphonium	Macrophage, mitochondria	Lipid Removal	MRI	[61]
	Gene therapy	LV / MNPs	/	Native endothelium	Overexpression of therapeutic genes eNOS and VEGF, improve vascular function	MRI	[71]
	Gene therapy	PEI‐SPION/siRNA	/	Macrophage	Induce siRNA‐mediated target gene silencing	MRI	[85]
	Interleukin 10 (IL10)	IL 10/IONP‐loaded cRGD	cRGD peptide	αvβ3‐integrin	Anti‐inflammatory	MRI	[72]
	Cell transplantation	USPION‐labeled EPCs	/	/	Repair endothelial damage and prevent atherosclerosis	MRI	[86]
	Phosphatidylserine	Phosphatidylserine‐presenting liposomes	Phosphatidylserine	Macrophage	Anti‐inflammatory	MRI	[87]

## 3 Iron Oxide Nanoparticles‐Based Theranostic Nanomedicine Utilizing Therapeutics‐Based Therapy

In the context of cancer treatment, chemotherapy typically refers to the use of chemical drugs for tumor management.^[^ ^88^ ^]^ However, in this review, the term “chemical therapy” is employed to distinguish it from cancer chemotherapy. This distinction encompasses the use of both organic chemical small molecule drugs and inorganic nanoparticles as therapeutic agents. The category of organic chemical small molecule drugs includes traditional therapeutic medicines and natural active compounds. Within the realm of atherosclerosis treatment, chemical therapy has gained extensive attention and significance. It now occupies a central role, particularly with the advent of drug nanocarriers and their rapid development and application. Chemical therapy, rooted in theranostic nanomedicine centered on IONPs, exhibits remarkable potential for addressing atherosclerosis.

### 3.1 Traditional Medicines

In clinical practice, drug therapy stands as one of the most prevalent methods for managing atherosclerosis, particularly playing a pivotal role in the early and intermediate stages of the disease. Various drugs find widespread clinical use in atherosclerosis treatment, including lipid‐lowering medications (e.g., simvastatin, rosuvastatin, atorvastatin, probucol, fenofibrate),^[^ ^89^ ^]^ antiplatelet agents (e.g., aspirin, cilostazol, dipyridamole, clopidogrel), vasodilators (e.g., hydralazine, sodium nitroprusside, captopril, diltiazem),^[^ ^90^ ^]^ and others such as antiangiogenic drugs, anti‐inflammatory agents, and immunosuppressants.^[^ ^91^ ^]^

#### 3.1.1 Immunosuppressants

**Figure 4:**
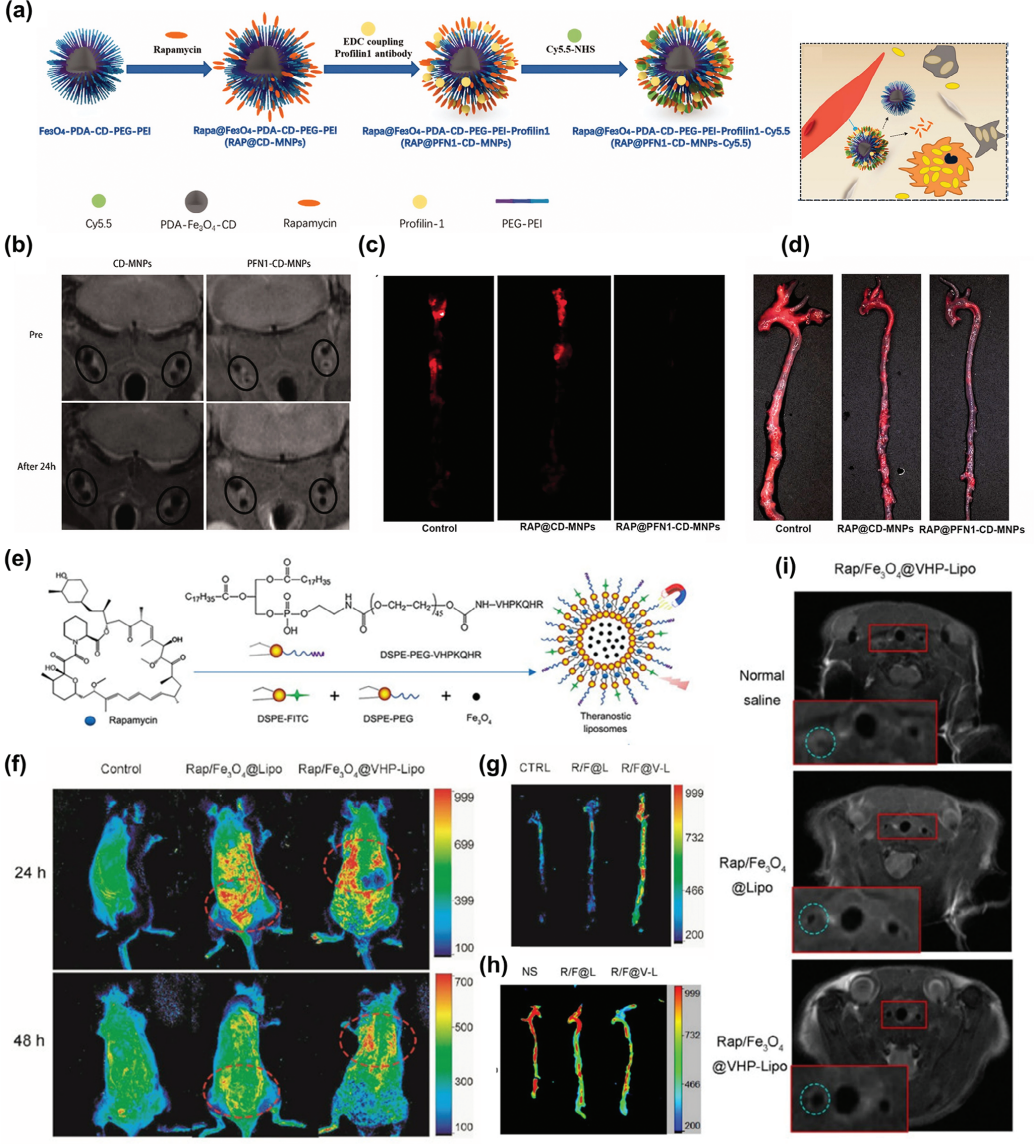
a) Schematic illustration of RAP@Fe_3_O_4_‐PDA‐CD‐PEG‐PEI‐Profilin‐1‐Cy5.5 nanoparticles. b) Representative in vivo MR images of ApoE^−/−^ mice with PFN1‐CD‐MNPs. c,d) NIRF of the aorta and general ORO staining of the carotid artery after 2 months of various treatments. Reproduced under terms of the CC‐BY license.^[^ ^38^ ^]^ Copyright 2019, The Authors, Published by Informa UK Limited, trading as Taylor & Francis Group. e) Schematic illustration of the synthesis of theranostic liposomes Rap/ Fe_3_O_4_@VHP‐Lipo by self‐assembly. f) The fluorescence distribution in vivo for mice with three different treatments. g) The fluorescence enrichment of the aorta from three different mice groups. h) The treatment effectiveness testing of fluorescence imaging for mice from three different groups injected with Rap/ Fe_3_O_4_@VHP‐Lipo for 2 months. i) MRI T2 mapping sequence imaging to detect the treatment effect of mice in three different groups with different treatments for 2 months. Reproduced under terms of the CC‐BY license.^[^ ^68^ ^]^ Copyright 2022, The Authors, Published by MDPI, Basel, Switzerland.

Rapamycin serves as an effective and specific inhibitor of the mammalian target of rapamycin (mTOR), disrupting the formation of foam cells by blocking mTOR activation, thereby impeding atherosclerosis progression.^[^ ^91d^ ^]^ To enhance the therapeutic efficacy, Zhang et al. ingeniously coupled the profilin‐1 antibody (PFN1, which actively targets vascular smooth muscle cells) with superparamagnetic iron oxide nanoparticles, subsequently incorporating rapamycin and Cy5.5. This innovative approach resulted in the development of dual‐mode imaging nanoparticles with therapeutic potential against atherosclerotic plaques (**Figure** **4**a). The study demonstrated that these nanoparticles could effectively locate atherosclerotic plaques through passive penetration and specific targeting of vascular smooth muscle cells in ApoE^−/−^ mice, observable through NIRF and in vivo MRI. The MRI images taken before and 24 h after NP injection (Figure 4b) highlighted a prominent increase in carotid artery wall thickness and plaque formation in the PFN1‐CD‐MNPs group compared to the control group. Subsequently, the nanoparticles exhibited a rapid release of rapamycin in the acidic atherosclerotic microenvironment, facilitated by pH‐sensitive cyclodextrin, effectively inhibiting atherosclerotic lesion development (Figure 4c, d).^[^ ^38^ ^]^

In a similar study, conducted by Chen et al., IONPs and rapamycin were encapsulated within liposomes and further modified with a fluorescent reagent. These nanoparticles were ingeniously equipped with a targeting peptide, VHPKQHR (VHP), enabling specific recognition and binding to VCAM‐1 on endothelial cells (Figure 4e). The results demonstrated the synthesized theranostic's efficacy in serving as an excellent label for MRI and fluorescence bimodal imaging of atherosclerosis, showcasing its promising potential for early diagnosis (Figure 4f,g). Furthermore, this theragnostic exhibited remarkable therapeutic efficacy against early‐stage atherosclerosis, achieving similar therapeutic effects with lower doses of rapamycin. This outcome was attributed to the enhanced stability and bioavailability of rapamycin facilitated by the nano‐drug delivery system (Figure 4h,i).^[^ ^68^ ^]^

#### 3.1.2 Antiangiogenic Drugs

Angiogenesis within the atherosclerotic plaque plays a pivotal role in plaque development. Neovascularization within the plaque leads to the leakage of erythrocytes and inflammatory mediators into the plaque's core, setting off a cascade of events that promote plaque instability.^[^ ^91b^ ^]^ Research has indicated that antiangiogenic therapy can effectively promote plaque stabilization and prevent further intraplaque hemorrhage by pruning and normalizing neovascularization within the plaque.^[^ ^92^ ^]^ In a study conducted by Winter et al. in 2006, an ανβ3‐targeted SPION was used in a rabbit model of atherosclerosis. This approach facilitated the detection of early atherosclerosis through MRI, the delivery of an antiangiogenic drug (fumagillin) for treatment, and the quantitative monitoring and assessment of neovascularization responses. The study's results revealed that after 7 days of a single treatment, rabbits treated with fumagillin‐loaded nanoparticles exhibited fewer microvessels in the aorta compared to control rabbits.^[^ ^39b^ ^]^

Subsequent research delved into the duration of the antiangiogenic effect of these nanoparticles. The findings indicated that the ανβ3‐targeted fumagillin nanoparticles could reduce neovascular signals by 50% to 75% within 1 week and maintain this effect for up to 3 weeks. Moreover, when researchers combined fumagillin with atorvastatin, they discovered that the addition of atorvastatin extended the antiangiogenic effect of fumagillin to more than 8 weeks. This achievement marked a significant and sustained impact on anti‐angiogenesis.^[^ ^39a^ ^]^ These results underscore the potential of targeted nanosystems, integrating imaging functionality and therapeutic drugs. Such systems not only enable the monitoring of targeted drug delivery to specific cells and tissues but also provide valuable insights into the patient's response to treatment. Given the prevalence of polypharmacy in current clinical applications, the feasibility of combining two or more drugs can be considered when designing nanosystems for drug delivery. This approach, drawing from clinical medication experience, may yield promising synergistic therapeutic effects. However, it is essential to explore and validate these novel combination therapeutic strategies through more in‐depth investigations.

#### 3.1.3 Anti‐Inflammatory Agents

At present, anti‐inflammatory agents have been proved promising in the treatment of atherosclerosis. However, few clinical anti‐inflammatory drugs have been applied in the theranostic of atherosclerosis based on IONPs. An illustrative study by Matuszak et al. involved the development of superparamagnetic iron oxide nanoparticles conjugated with dexamethasone phosphate (SPIONs‐DEAX, an anti‐inflammatory glucocorticoid) and its assessment in a rabbit model of atherosclerosis. Nevertheless, SPIONs‐DEAX did not yield the anticipated anti‐inflammatory therapeutic effects. But noticeably, the data demonstrated their biocompatibility and precise targeting capabilities.^[^ ^73^ ^]^ The study harnessed the superparamagnetic properties of IONPs to guide drug enrichment at the plaque site using a magnetic field—a technique known as magnetically controlled drug targeting (MCDT).^[^ ^93^ ^]^ The successful accumulation of these nanoparticles within the area of arterial injury in vivo provided valuable evidence that magnetically‐targeted drug delivery could serve as an effective platform for transporting drugs to afflicted arteries. Consequently, maximizing the effectiveness and controlled mobility inherent to magnetically controlled drug delivery becomes paramount, emphasizing the need for strategic design in nano‐drug delivery systems applied to atherosclerosis theranostics. Regarding the anti‐inflammatory effect of atherosclerosis, many other studies, except clinical anti‐inflammatory drugs, have been reported and shown good prospects. However, the related exploration based on clinical anti‐inflammatory drugs with IONPs’ nanotheranostic is also expected.

### 3.2 Natural Medicines

While conventional drugs like statins and rapamycin are widely used and effective in preventing and treating atherosclerosis, mounting evidence suggests that long‐term usage of these drugs may lead to serious adverse reactions. Therefore, there is an urgent need to discover alternative drugs with a higher safety profile.^[^ ^94^ ^]^ Natural medicines have gained increasing attention in recent years due to their excellent biosafety and broad therapeutic effects against various diseases.^[^ ^95^ ^]^ Importantly, numerous scientific studies have highlighted the significant potential of natural medicines in treating atherosclerosis by exerting anti‐inflammatory, antioxidant, and anti‐apoptotic effects on vascular endothelial cells, etc. Consequently, several IONP‐based theranostic platforms for delivering natural medicines have been developed and demonstrated remarkable anti‐atherosclerotic effects.

#### 3.2.1 Inhibit Platelet Activation and Aggregation

**Figure 5:**
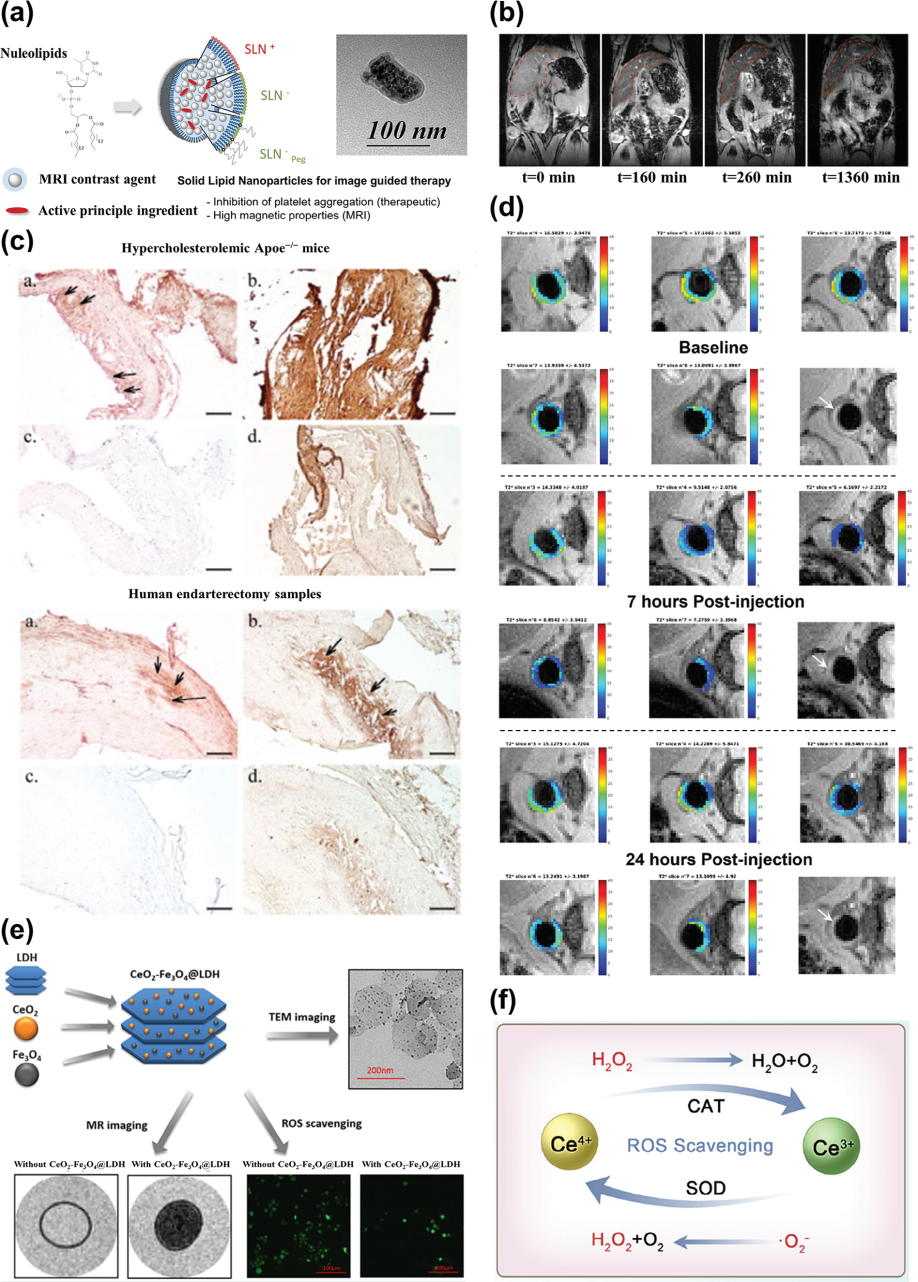
a) Schematic illustration of prostacyclin‐loaded SLN for image‐guided Therapy. Reproduced with permission.^[^ ^62^ ^]^ Copyright 2016, American Chemical Society. b–d) Atheroma targeting and in vivo MRI ability of the nano‐emulsion platform modified with a fully human scFv‐Fc antibody. Reproduced under terms of the CC‐BY license.^[^ ^74^ ^]^ Copyright 2021, The Authors, Published by MDPI, Basel, Switzerland. e) Schematic diagram of the structure and theranostic function of CeO_2_‐ Fe_3_O_4_@LDH nanocomposites. Reproduced with permission.^[^ ^63^ ^]^ Copyright 2019, American Chemical Society. f) Mechanism of ROS Scavenging by CeO_2_ NPs. Reproduced with permission.^[^ ^104^ ^]^ Copyright 2023, American Chemical Society.

One such example is prostacyclin (PGI2), a naturally occurring bioactive lipid known for its pharmacological properties in inhibiting platelet aggregation. Multiple studies have illustrated the atheroprotective potential of PGI2, attributed to its ability to inhibit platelet activation and aggregation, as well as regulate lipid peroxidation.^[^ ^96^ ^]^ For instance, Oumzil et al. introduced a novel approach involving nucleoside‐lipid insertion to synthesize stable solid lipid nanoparticles (SLN) loaded with iron oxide particles and natural active drugs, as depicted in **Figure** **5**a. Their investigation evaluated the inhibitory effects of prostacyclin‐loaded SLN on platelet activation and aggregation, offering a promising avenue for the development of innovative theranostic tools targeting atherosclerosis.^[^ ^62^ ^]^

#### 3.2.2 Anti‐Inflammatory and Antioxidant

Besides their antiplatelet effects, most of the reported natural medicines possess anti‐inflammatory and antioxidant properties, thereby exhibiting multi‐targeted anti‐atherosclerotic properties. These natural medicines encompass a diverse array of compounds, including alkaloids, glycosides, flavonoids, terpenes, quinones, and polyphenols.^[^ ^97^ ^]^ Among them, α‐tocopherol, a highly prevalent and potent form of vitamin E found in nature, is known for its exceptional natural antioxidant properties.^[^ ^98^ ^]^ A noteworthy study by Bonnet et al. involved the design of oil‐in‐water nano‐emulsions (NEs) loaded with α‐tocopherol and SPIONs. These NEs were further modified with polyethylene glycol and functionalized with the target ligand P3, a fully human scFv‐Fc antibody. This innovative approach led to the development of a multifunctional therapeutic and diagnostic tool tailored for atherosclerosis. The outcomes of this study demonstrated the nanoparticles' capability for in vivo targeting of atherosclerotic plaques in ApoE^−/−^ mice. Moreover, the in vivo behavior of these nanoparticles could be accurately monitored over time, offering a promising avenue for molecular imaging to guide more personalized approaches to atherosclerosis treatment, as depicted in Figure 5b–d. The research solely examined the nanoparticles' capacity to scavenge reactive oxygen species in vitro, without assessing their effectiveness in treating atherosclerosis in vivo.^[^ ^74^ ^]^

Polyphenols, like protocatechuic acid (PCA), which is a major anti‐inflammatory metabolite of complex polyphenols, often suffer from limitations such as poor water solubility, low bioavailability, and low stability, hindering their application as therapeutic agents.^[^ ^99^ ^]^ To address this issue, Anghelache et al. developed dextran‐coated magnetic nanoparticles (MNPs) loaded with PCA. These MNPs could not only utilize their magnetic properties to guide the nanomedicine to the target but also enhance the anti‐inflammatory efficacy of PCA by improving its cellular uptake and internalization.^[^ ^54a^ ^]^

#### 3.2.3 Anti‐Atherosclerosis Effect of Natural Chemotherapeutic Agents

Natural medicines often exhibit multifunctionality and can be employed for a wide range of diseases. Several natural chemotherapeutic agents, owing to their antioxidant capacity, can serve as anti‐atherosclerotic agents. For instance, curcumin is one such agent. In a recent study, Qiu et al. designed a multifunctional nanoparticle system loaded with curcumin for the treatment of atherosclerosis. This research encapsulated SPIONs and curcumin within amphiphilic polymer DSPE‐PEG. Subsequently, they coupled VCAM‐1 and Cy5.5 to this system via amidation reactions, forming a theranostic nanoplatform with dual‐modal imaging capabilities (MRI and fluorescence imaging) and targeted treatment for atherosclerosis.^[^ ^75^ ^]^ Furthermore, paclitaxel, a natural anticancer drug known for its antiproliferative and immunomodulatory effects, has shown effectiveness in alleviating atherosclerotic lesions by inhibiting macrophage migration, vascular smooth muscle cell hyperproliferation, and intimal invasion in a rabbit model of atherosclerosis.^[^ ^100^ ^]^ Thus, Dong et al. constructed a theranostic nanoparticle (UP‐NP‐C11) loaded with ultrasmall superparamagnetic iron oxide (USPIO) and paclitaxel, using a polymer‐lipid hybrid. They also conjugated it with C11, a polypeptide targeting collagen IV, for simultaneous MRI and atherosclerosis treatment. The results revealed that UP‐NP‐C11 exhibited superior in vivo MRI capabilities and more significant therapeutic effects in rabbit atherosclerosis compared to UP‐NP and commercially available USPIO + paclitaxel.^[^ ^45c^ ^]^ Collectively, the newly designed IONPs‐based nanosystems can serve not only as in vivo imaging probes but also as carriers for delivering natural medicines to inflammatory tissues, thereby increasing bioavailability and treatment efficiency. This approach offers a promising avenue for the molecular imaging and targeted therapy of atherosclerosis, attributed to its established specificity and high safety profile. Furthermore, it can potentially be applied to the treatment of other inflammatory diseases.

### 3.3 Inorganic Nanoparticles

Excessive production of reactive oxygen species (ROS) stands as a prominent contributor to the progression of various inflammatory diseases, including atherosclerosis.^[^ ^101^ ^]^ Thus, targeting ROS, mitigating oxidative stress, and modifying the inflammatory microenvironment within plaques emerge as crucial strategies in atherosclerosis treatment. An array of anti‐ROS drugs, such as natural antioxidants, has been extensively investigated and applied, as discussed in the preceding section. Additionally, numerous metal nanozymes exhibit exceptional superoxide dismutase mimetic activity and catalase mimetic activity, effectively scavenging ROS during chronic inflammation.^[^ ^102^ ^]^

Presently, the ROS‐scavenging potential of cerium oxide (CeO_2_) nanoparticles (NPs) in the context of inflammatory diseases has garnered substantial support from several studies.^[^ ^103^ ^]^ Within the domain of atherosclerosis, researchers have harnessed the synergy of CeO_2_ NPs and superparamagnetic iron oxide nanoparticles to engineer a variety of versatile theranostic nanoplatforms. One such innovation was introduced by Wu et al., who synthesized a novel theranostic nanoparticle comprising an IONPs core and a CeO_2_ NPs shell. This hybrid nanoparticle offered both diagnostic capabilities, through IONPs facilitating MRI, and therapeutic functionalities, via CeO_2_ NPs imparting anti‐ROS effects. Figure 5f illustrates the potential mechanism underpinning the remarkable regenerative antioxidant properties of CeO_2_ NPs: the conversion between Ce^3+^ and Ce^4+^ on the nanoparticle surface allows them to scavenge superoxide radicals.^[^ ^104^ ^]^ Moreover, it has been established that the Ce^3+^/Ce^4+^ ratio significantly influences the anti‐ROS efficacy of the nanoparticles.^[^ ^76^ ^]^ Furthermore, various reliable multifunctional nanocomposites have been devised to deliver both IONPs and CeO_2_ NPs, boasting extended in vivo circulation times and providing abundant attachment sites for further modifications, such as the addition of targeting ligands. Liu et al. selected the layered double hydroxides (LDHs), biodegradable 2D layered nanomaterials with exceptional biocompatibility, to serve as a carrier. They ingeniously loaded both IONPs and CeO_2_ NPs onto the surface of LDHs through electrostatic interactions, as illustrated in Figure 5e. The outcome demonstrated that these nanocarriers could extend the half‐life of CeO_2_ NPs and IONPs in vivo without compromising the antioxidant capacity of CeO_2_ NPs or the efficacy of MRI signal detection in macrophages.^[^ ^63^ ^]^

In a separate investigation, Wu et al. embarked on the development of chitosan nanococktail theranostic materials, meticulously assembling IONPs and CeO_2_ NPs with chitosan. This assembly was achieved through two distinct mechanisms: electrostatic self‐assembly and ionic gelation.^[^ ^77^ ^]^ A standout feature of this study is its adept utilization of independently synthesized and modified IONPs and CeO_2_ NPs as two modular components within a versatile theranostic nanoplatform. This modular design affords flexibility for adapting the nanoplatform to future applications and requirements while permitting facile adjustment of the loadings for each module. Consequently, this approach offers a promising blueprint for crafting a theranostic nanoplatform tailored for delivering inorganic nanoparticles with inherent antioxidant capabilities alongside imaging agents. Furthermore, the relatively straightforward synthesis process of the nanococktail theranostic nanoplatform lends itself to advantages in terms of scalability and clinical translation Nevertheless, it is crucial to underscore that the MRI and anti‐inflammatory therapeutic effects observed in these nanoplatforms, which integrate IONPs and CeO_2_ NPs, necessitate validation through further in vivo studies employing atherosclerosis models. Moreover, the potential augmentation of targeted therapeutic effects, enhancement of detection sensitivity, and optimization of pharmacokinetic profiles can be explored by incorporating targeting molecules into these nanoplatforms to refine their performance. Furthermore, as for the above theranostic nanosystem, the advantage of inorganic nanoparticles lies in their flexibility regarding structure, size, and shape regulation, which allows for precise design and optimization.^[^ ^105^ ^]^ The metal nanozymes can obtain superior catalytic efficiency through regulating synthetic methods, while IONPs can also acquire more suitable and excellent properties according to the requirement of the application.

## 4 Iron Oxide Nanoparticle‐Based Theranostic Nanomedicine Using Physical Stimulation Therapy

Physical stimulation therapy encompasses the application of specific physical stimuli externally, such as light, magnetic fields, ultrasound, etc. These stimuli act on materials possessing distinct physicochemical properties, including photosensitizers, photothermal materials, magnetothermal materials, and sonosensitizers, which accumulate at the disease site within the body. The goal is to induce therapeutic effects, such as the generation of ROS for cell eradication, the precise ablation of diseased tissues through controlled temperature elevation, and the initiation of programmed cell death. Currently, physical stimulation therapy stands as an indispensable modality in atherosclerosis treatment, with diverse strategies emerging to harness its potential. This section offers an overview of theranostic nanoplatforms based on IONPs employing physical stimulation therapy for addressing atherosclerosis. These strategies encompass photodynamic therapy, photothermal therapy, magnetic hyperthermia (MHT), and ultrasound therapy.

### 4.1 Photodynamic Therapy

**Figure 6:**
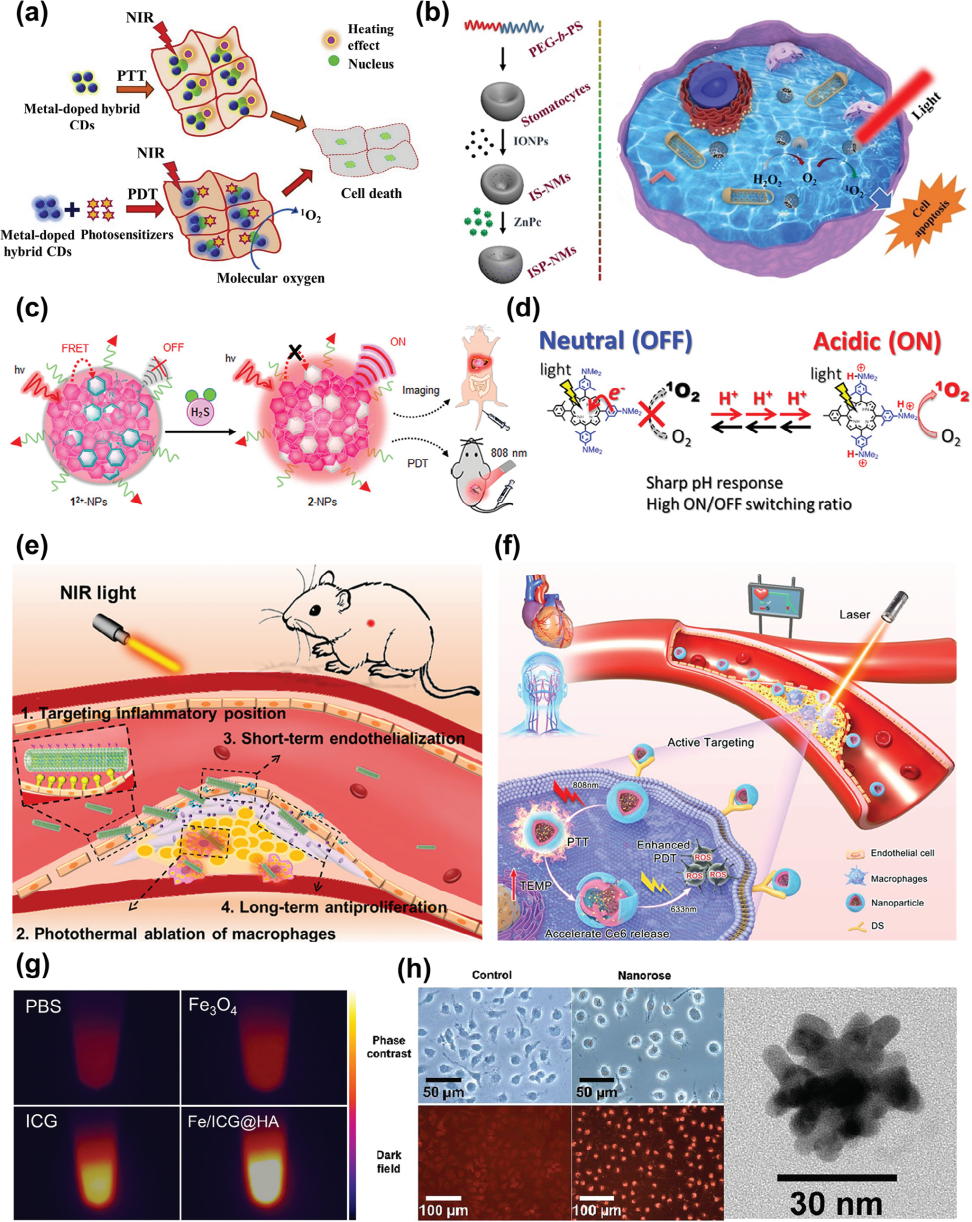
a) Schematic illustration of phototherapy. Reproduced with permission.^[^ ^106^ ^]^ Copyright 2020, Elsevier B.V. b) Schematic diagram of cell death by PDT. Reproduced with permission.^[^ ^111^ ^]^ Copyright 2020, Elsevier B.V. c) Activatable photosensitization responds to H_2_S. Reproduced with permission.^[^ ^123^ ^]^ Copyright 2018, American Chemical Society. d) Activatable photosensitization responds to pH change. Reproduced with permission.^[^ ^124^ ^]^ Copyright 2016, American Chemical Society. e) Schematic illustration of PTT in the treatment of atherosclerosis. Reproduced with permission.^[^ ^125^ ^]^ Copyright 2021, American Chemical Society. f) Schematic illustration of sequential photothermal/photodynamic ablation for activated macrophages. Reproduced with permission.^[^ ^126^ ^]^ Copyright 2021, American Chemical Society. g) Near‐infrared thermal imaging images of PBS, ICG, Fe_3_O_4_, and Fe/ICG@HA after irradiation with an 808 nm laser. Reproduced with permission.^[^ ^127^ ^]^ Copyright 2022, American Chemical Society. h) Small multifunctional nanoclusters and their capability of PTT. Reproduced with permission.^[^ ^64^ ^]^ Copyright 2009, American Chemical Society.

Phototherapy, a burgeoning medical treatment characterized by spatiotemporal selectivity and non‐invasiveness, encompasses both photodynamic therapy (PDT) and photothermal therapy (PTT) (**Figure** **6**a).^[^ ^106^ ^]^ Nowadays, PDT finds extensive applications in cancer,^[^ ^107^ ^]^ antimicrobial,^[^ ^108^ ^]^ anti‐inflammatory,^[^ ^109^ ^]^ and anti‐atherosclerotic therapies.^[^ ^110^ ^]^ The basic therapeutic mechanism of PDT unfolds as follows: a photosensitizer, upon accumulation within specific tissues or cells, undergoes alteration when exposed to an appropriate light wavelength, typically near‐infrared (NIR). This interaction triggers a reaction with oxygen molecules, generating reactive oxygen species (ROS) like hydroxyl radicals (·OH), superoxide radical anions (·O_2_−), and singlet oxygen (^1^O_2_). Ultimately, these ROS induce cell apoptosis, cell necrosis, or autophagic cell death via oxidative damage,^[^ ^107a^ ^]^ as depicted in Figure 6b.^[^ ^111^ ^]^

The theranostic nanosystems of atherosclerosis combining IONPs with PDT have been reported. Among them, the principal role of PDT in atherosclerosis therapy involves the generation of ROS, leading to extensive cell death in plaque regions where photosensitizers are sufficiently concentrated,^[^ ^110a^ ^]^ while IONPs usually function as imaging agents and/or nanocarriers. Precise delivery of adequate photosensitizer doses to the plaque site is crucial for achieving favorable treatment outcomes in PDT for atherosclerosis. Conjugating photosensitizers with nanocarriers or targeting agents can substantially enhance therapeutic effects and reduce side effects arising from nonspecific photosensitizer localization. In 2006, McCarthy et al. developed a nanosystem with combined diagnostic and therapeutic functionalities by covalently attaching a potent photosensitizer, 5‐(4‐carboxyphenyl)−10,15,20‐triphenyl‐2,3‐dihydroxychlorin (TPC), to crosslinked dextran‐coated iron oxide (CLIO) nanoparticles possessing macrophage‐targeting properties. They also incorporated a near‐infrared fluorophore into the system. This nanoplatform was capable of MRI and near‐infrared fluorescence imaging, exhibited a high macrophage uptake rate, and effectively induced apoptosis in mouse and human macrophages under light exposure while demonstrating no toxicity in the absence of light.^[^ ^78^ ^]^

The performance of the photosensitizer also significantly influences PDT's therapeutic effectiveness. As for the nanoagent, CLIO‐TPC, the conjugation of the photosensitizer to the polymer‐coated nanoparticles faced challenges due to its nonpolar nature, resulting in reduced stability. To enhance the overall phototoxicity and stability of the nanoagent, McCarthy et al. developed a novel hydrophilic photosensitizer (THPC) based on meso‐tetra(m‐hydroxyphenyl) chlorin, which was then coupled with CLIO nanoparticles. This new photosensitizer exhibited a threefold higher loading on CLIO nanoparticles compared to TPC. Significantly, the synthesized nanoagent remained stable in suspension for up to 1 year. Moreover, a hind paw edema model experiment demonstrated that CLIO‐THPC had lower skin phototoxicity compared to the conventional photosensitizer chlorin e6. The imaging and therapeutic effects of the nanoagent were validated in cellular experiments and studies on ApoE^−/−^ mice. RAW 264.7 cells that engulfed CLIO‐THPC underwent substantial abnormal cell death upon irradiation with a 650 nm laser. In ApoE^−/−^ mice studies, the results indicated that CLIO‐THPC could efficiently localize to carotid atherosclerotic plaque sites in vivo and induced significant apoptosis of macrophages. This study underscores the potential of PDT for achieving targeted focal macrophage ablation to stabilize plaques. It also highlights the substantial value of combining PDT with multimodality imaging detection, leveraging IONPs and other imaging agents to create a theranostic nanoplatform. However, it's worth noting that this study utilized an exposed carotid artery in a murine model for imaging and treatment, which is constrained by the depth of light penetration. Therefore, there is an expectation to identify longer‐wavelength photosensitizers (>750 nm) to improve light penetration into tissues for noninvasive detection and PDT in deeper vascular systems.^[^ ^79^ ^]^

Alongside the photosensitizers mentioned above, IONPs can connect with other photosensitizers like indocyanine green (ICG) to obtain supreme photosensitive performance (Figure 6g). Alongside targeting strategies, the concept of activatable photosensitizers has recently emerged as a means to minimize damage to surrounding healthy tissues during PDT (Figure 6c,d). This approach has seen extensive exploration in the context of tumor PDT. For instance, certain photosensitizers are administered into the body in an inactive form. Upon reaching the tumor site, they are activated by specific conditions, such as proteolytic enzymes or pH conditions present within the tumor microenvironment, before they can exert their normal functions.^[^ ^112^ ^]^ In the treatment of atherosclerosis, there has been significant research into designing responsive therapeutic strategies tailored to the unique physiological environment at the plaque site. These strategies encompass various stimuli‐responsive approaches, including responses to ROS, enzymes (e.g., matrix metalloproteinases, hyaluronidases, cathepsin), pH changes, and shear stress.^[^ ^5^ ^]^ Consequently, greater attention can be directed toward developing innovative activatable photosensitizer molecules designed to respond to the specific environmental conditions of atherosclerotic plaques. This can lead to safer and more effective PDT outcomes.

### 4.2 Photothermal Therapy

Much like PDT, PTT presents an appealing approach for treating atherosclerosis, such as the ablation of inflammatory macrophages within plaques, and notable research progress has been achieved (Figure 6e).^[^ ^113^ ^]^ In atherosclerosis, this method involves the delivery of a photothermal agent to a plaque site, where the agent absorbs the laser energy and transforms it into thermal energy, resulting in localized heating and cell ablation(Figure 6a).^[^ ^8^, ^106^ ^]^ Meanwhile, the therapeutic effect is confined solely to the disease area where the photothermal agent is present and where the laser is focused. Consequently, PTT offers a high degree of spatiotemporal selectivity.^[^ ^114^ ^]^ Moreover, the radiation wavelength used to excite the photothermal material is longer than PDT, enabling deeper penetration into biological tissues while causing less harm to neighboring cells and tissues.

#### 4.2.1 IONPs Modified with Photothermal Materials Serving as Photothermal Agents

IONPs can be combined with various photothermal materials to create nanoparticles that possess both magnetic and NIR absorption properties. Examples of photothermal materials include gold‐based nanomaterials,^[^ ^64^, ^115^ ^]^ molybdenum sulfide,^[^ ^116^ ^]^ carbon‐based materials,^[^ ^117^ ^]^ Prussian blue,^[^ ^118^ ^]^ and copper sulfide.^[^ ^119^ ^]^ For instance, Ma et al. developed primary iron oxide nanoparticles with a thin gold coating and assembled them into stable ≈30 nm nanoclusters using dextran as a stabilizer. These nanoclusters exhibited MRI and NIRF detection capabilities, targeted macrophages, and enabled efficient PTT for atherosclerosis. Their small size and dextran coating facilitated uptake by dextran receptor‐containing macrophages, allowing for high NIRF contrast in the aortic imaging of atherosclerotic rabbits. Moreover, results from both cellular experiments and animal models indicated that macrophages in atherosclerotic plaques could selectively take up the theranostic nanoclusters and be eliminated by subsequent laser irradiation (Figure 6h).^[^ ^64^ ^]^ However, further in‐depth and comprehensive experiments are needed to fully evaluate and confirm the therapeutic effects of these nanoclusters in vivo.

#### 4.2.2 IONPs Serving as Photothermal Agents

IONPs themselves can serve as photothermal agents without requiring modification with gold or other photothermal materials. The photothermal conversion capability of IONPs has been extensively documented, and its application in PTT has been explored. Shen et al. developed Fe_3_O_4_ nanoparticles stabilized with carboxymethyl chitosan and demonstrated their exceptional photothermal effect and ability to ablate disease cells through photothermal therapy. Under NIR laser irradiation, these Fe_3_O_4_ nanoparticles rapidly generated heat, elevating the temperature from ≈25 °C to ≈80 °C in just ≈5 min. Importantly, the difference in their NIR photothermal heating capability compared to that of HAuNS under the same conditions was not significantly pronounced.^[^ ^120^ ^]^ Additionally, Chu et al. synthesized various shapes of Fe_3_O_4_ magnetic nanoparticles, including spherical, hexagonal, and wire‐like nanoparticles, all of which exhibited robust photothermal effects induced by NIR laser irradiation.^[^ ^121^ ^]^ Furthermore, Shen et al. conducted a study that investigated the impact of the aggregation state of Fe_3_O_4_ nanoparticles on their photothermal effect. It was found that clustered Fe_3_O_4_ nanoparticles exhibited higher NIR light absorption compared to individual Fe_3_O_4_ nanoparticles, resulting in more efficient temperature elevation and enhanced cell eradication.^[^ ^122^ ^]^

In the context of atherosclerosis, ferrite nanoparticles, a subtype of IONPs extensively studied for their photothermal properties and mechanisms, have been employed to develop theranostic nanomedicine due to their MRI capabilities and suitability for PTT applications. Yang et al. engineered a manganese ferrite (MnFe_2_O_4_)‐encapsulated nanoparticle with multimodal imaging‐guided functionality and stable photothermal performance for targeted imaging and therapy of neovascularization at atherosclerotic plaque sites. This theranostic nanomedicine featured 3 nm MnFe_2_O_4_ NPs loaded within a biocompatible PLGA polymer, serving dual roles as an imaging agent for MRI T1 and photoacoustic imaging (PAI), as well as a therapeutic agent for PTT. Moreover, an anti‐VEGFR2 antibody, ramucirumab (Ram), was conjugated to the surface of poly (lactic‐*co*‐glycolic acid (PLGA) shells to specifically target atherosclerotic neovasculature. Furthermore, liquid perfluorohexane (PFH), a phase‐transition material capable of generating gas microbubbles via optical droplet vaporization, was included to enable ultrasound (US) imaging in vivo. Thus, the nanomedicine enabled multimodal imaging using MRI, PAI, and US imaging. In a study conducted using a rabbit‐advanced atherosclerotic plaque model, the theranostic nanomedicine exhibited remarkable multimodal imaging capabilities for rabbit femoral plaques, with an evident targeting effect. Meanwhile, the results of experiments in vivo confirmed the significant efficacy of PTT guided by PFH@PLGA/Mn Fe_2_O_4_‐Ram NPs. This included the induction of neovascular endothelial cell apoptosis, a reduction in neovessel density, and ultimately the stabilization of rabbit plaques. Importantly, the results also verified its substantial therapeutic biosafety. Collectively, these findings suggest that PFH@PLGA/MnFe_2_O_4_‐Ram is a promising photothermal nanoagent for safely and effectively inhibiting angiogenesis in atherosclerosis.^[^ ^80^ ^]^ However, it is essential to address the challenge of limited tissue penetration of light irradiation in PTT, especially for deep‐seated vascular lesions. Therefore, further exploration and development of innovative strategies are needed to overcome this limitation and enhance the efficacy of PTT in treating atherosclerosis. Moreover, due to the similarity between PDT and PTT, combining these two strategies for the treatment of atherosclerotic plaque is also a way to improve the therapeutic effect at present (Figure 6f).

### 4.3 Magnetic Hyperthermia

Unlike photothermal therapy, which relies on light stimulation, magnetic hyperthermia (MHT) harnesses the conversion of externally applied high‐frequency magnetic fields into thermal energy using magnetic nanomaterials for disease treatment. MHT offers advantages such as deep tissue penetration and reduced toxicity, making it an appealing therapeutic option.^[^ ^128^ ^]^ Notably, IONPs, which are clinically approved MRI contrast agents, are commonly employed as hyperthermia agents in MHT. This dual functionality allows IONPs to provide diagnostic insights by enabling their visualization and distribution tracking in vivo via MRI, thereby facilitating guided MHT.^[^ ^129^ ^]^

IONP‐based multifunctional nanoplatforms have shown significant promise in advancing the treatment of atherosclerosis, with an intriguing study conducted by Chandramouli et al. offering notable insights. In this study, superparamagnetic iron oxide nanoparticles (SPIONs) were actively targeted to atherosclerotic plaques using an external magnetic field. Under the influence of an alternating‐current magnetic field, these SPIONs rapidly oscillated, generating substantial heat. This dual mechanism led to the reduction of plaque hardness due to transient thermal expansion and facilitated the removal of atherosclerotic plaque from the vessel wall by controlled SPION‐induced abrasion.^[^ ^81^ ^]^

**Figure 7:**
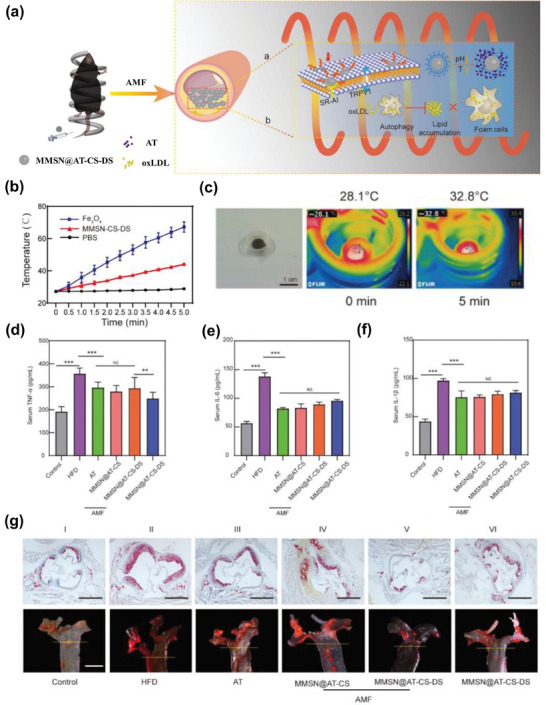
a) Schematic illustration of MMSN@AT‐CS‐DS NPs for multi‐effective treatment of atherosclerosis. b,c) Characterization of MMSN‐CS‐DS in magnetocaloric effect, including heating curves (b) and in vitro temperature raising (c). d–f) The levels of TNF‐α, IL‐6, and IL‐1β in blood serum from different mouse groups. g) Therapeutic efficacy in atherosclerotic mouse. ORO staining of the aortic roots and aortic arches from different mouse groups. Reproduced with permission.^[^ ^82^ ^]^ Copyright 2022, Elsevier B.V.

However, it is essential to recognize that a single therapeutic modality may often be insufficient to effectively address atherosclerosis. Therefore, combining therapies, a highly effective strategy in the current treatment landscape of various diseases should be considered.^[^ ^130^ ^]^ IONPs, when coupled with organic or inorganic compounds, have demonstrated remarkable outcomes in terms of stability, targeting capability, biocompatibility, and imaging. As such, they are recognized as promising nanomaterials for mediating drug delivery. A recent study conducted by Fang et al. exemplifies the potential of IONP‐based nanosystems for achieving multi‐effective treatment of atherosclerosis by combining magnetic hyperthermia and chemical therapy (**Figure** **7**). In this study, a dual‐targeted nanoplatform (MMSN@AT‐CS‐DS) was designed to offer diagnostic and therapeutic effects, as well as MRI capabilities. The core of the nanosystem consisted of magnetic mesoporous silica nanoparticles (MMSN), which served as therapeutic agents mediating magneto‐thermal conversion and inducing macrophage autophagy in atherosclerotic plaques. This magneto‐thermal effect was achieved through local heating, opening the thermosensitive cation channel TRPV1 in macrophages and triggering protective autophagy. Consequently, this led to a reduction in lipoprotein accumulation and the deceleration of atherosclerosis progression. Furthermore, the MMSN core was modified with chitosan and dextran, serving as a nanocarrier (MMSN‐CS‐DS) loaded with atorvastatin, a widely‐used lipid‐lowering drug. The release of atorvastatin was contingent on specific conditions, including elevated temperature and low pH, which are typically found in the plaque microenvironment. Finally, the MMSN core had the additional function of MRI, providing valuable information to guide magnetic hyperthermia therapy. The targeting strategy of the nanoplatform was dual‐responsive, involving biological targeting (SR‐A, recognized by DS on the surface of activated macrophages) and pH‐targeting (leveraging the low pH environment within plaques). The study conducted in the ApoE^−/−^ mice model demonstrated impressive results, with the MMSN@AT‐CS‐DS + AMF group showing the most significant therapeutic efficacy against atherosclerosis. This work by Fang et al. underscores the tremendous potential of IONPs in the theranostics of atherosclerosis.^[^ ^82^ ^]^

**Figure 8:**
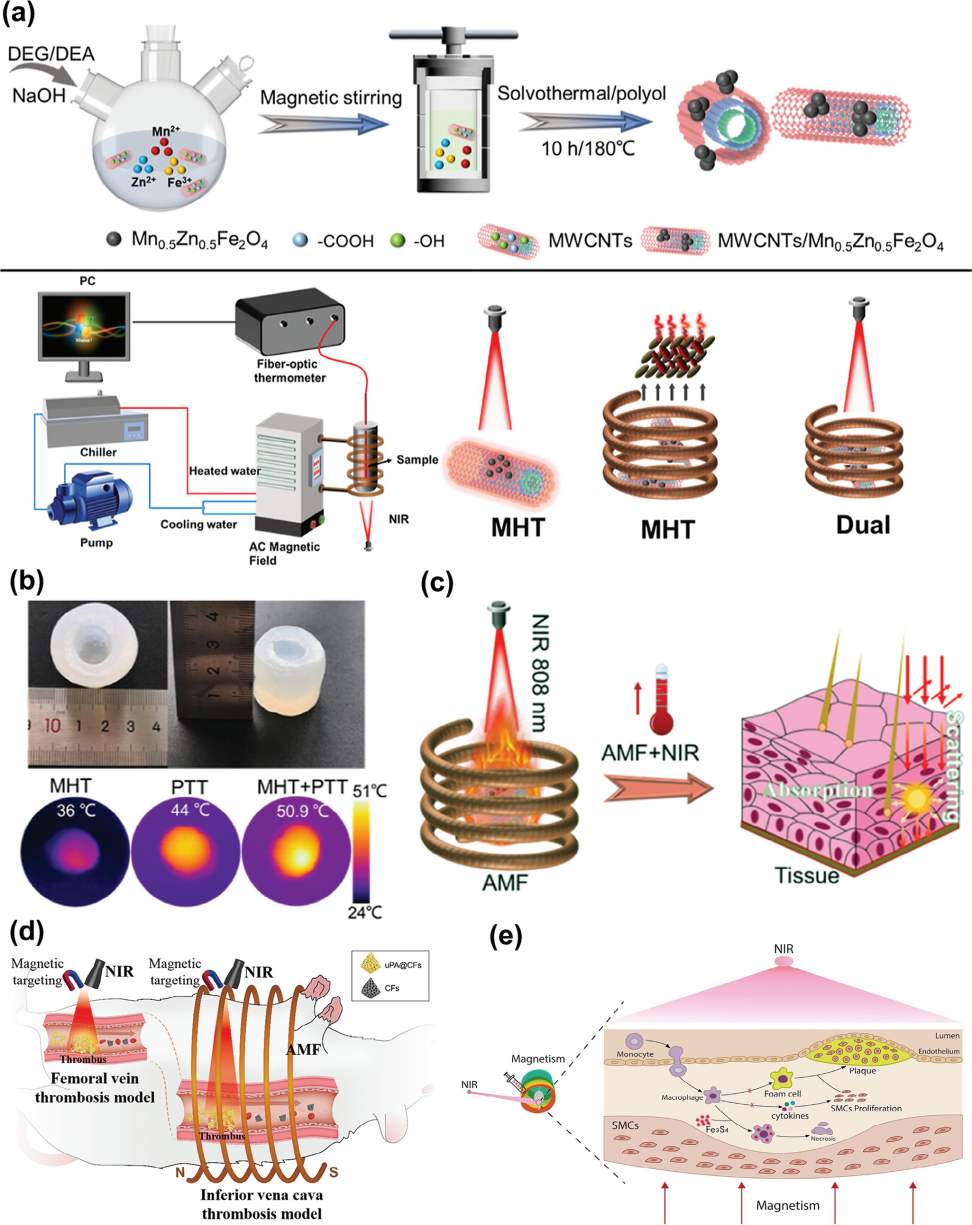
a) Schematic illustration of the synthesis process and measurement setup of Magneto‐Photothermal hybrids based on IONPs. b) Comparison of thermal properties of MHT, PTT, and DUAL. c) Schematic illustration of Magneto‐Photothermal therapy. Reproduced with permission.^[^ ^133a^ ^]^ Copyright 2023, American Chemical Society. d) Schematic diagram of MHT and PTT applying Iron‐based NPs in thrombolytic therapy. Reproduced with permission.^[^ ^133c^ ^]^ Copyright 2021, American Chemical Society. e) Schematic illustration of MHT and PTT based on Iron‐based nanoparticles in arterial inflammation therapy. Reproduced under terms of the CC‐BY license.^[^ ^133b^ ^]^ Copyright 2019, The Authors, Published by Elsevier Ltd.

Beyond their use in combination with chemotherapy, IONPs can serve as versatile tools for both photothermal therapy (PTT) and magnetic hyperthermia (MHT). When subjected to the dual stimulation of an alternating magnetic field and near‐infrared (NIR) laser irradiation, their heating effect can reach two to five times that achieved through single stimulation, yielding unprecedented heating capabilities.^[^ ^131^ ^]^ PTT and MHT, as the primary modalities of thermotherapy, each have their own set of advantages and limitations. For instance, PTT is constrained by the limited penetration ability of laser light and is not well‐suited for treating deep‐seated lesions. In contrast, MHT boasts excellent tissue penetration capabilities and can be employed without depth constraints. However, MHT's heat production rate falls significantly behind that of PTT.^[^ ^132^ ^]^ Therefore, the strategic combination of MHT and PTT effectively complements their strengths and weaknesses, yielding superior therapeutic outcomes. To put it differently, this combination allows for the achievement of equivalent or even more robust therapeutic effects compared to each modality on its own, all while minimizing damage to healthy tissues, owing to lower iron doses, manageable magnetic fields, and laser power doses. Recent studies have developed magneto‐photothermal hybrids based on IONPs or Iron‐based nanoparticles used for the ablation of diseased tissues, some of which have been researched in cardiovascular diseases (**Figure** **8**).^[^ ^133^ ^]^ Nanoparticle‐based combination therapy involving PTT and MHT has demonstrated remarkable potential in addressing deep‐seated arterial inflammation, showcasing superior efficacy in eradicating inflammatory infiltrating macrophages, and subsequently inhibiting the formation of atherosclerotic lesions and arterial stenosis.^[^ ^134^ ^]^ It is anticipated that future theranostic nanoplatforms based on IONPs will increasingly adopt this strategy for treating atherosclerosis.

### 4.4 Ultrasound Therapy

Ultrasound is widely regarded as a safe external physical stimulus, characterized by its non‐invasiveness, lack of toxicity, and absence of radiation contamination.^[^ ^135^ ^]^ Besides ultrasound imaging, noninvasive ultrasound therapy, attributed to its superior tissue penetration capabilities compared to light‐based stimulation and its capacity to minimize adverse effects on surrounding healthy tissues, has undergone extensive basic and preclinical research in the context of cardiovascular diseases, spanning ischemic heart disease, heart failure, myocarditis, arrhythmias, and hypertension, resulting in notable advancements.^[^ ^136^ ^]^ Among these, two ultrasound‐targeted therapies—sonothrombolysis and ultrasound‐mediated flow augmentation or sonoperfusion, have progressed into clinical trials.^[^ ^137^ ^]^ In atherosclerosis, the theranostic nanosystems of IONPs associated with ultrasound therapy have been investigated and reported.

#### 4.4.1 Ultrasound Therapy through Acoustic Droplet Vaporization Effect

Acoustic droplet vaporization (ADV), one of the unique physical effects contributing to the therapeutic utility of ultrasound in vivo,^[^ ^138^ ^]^ involves the process where nanoscale droplets experience regular variations in internal pressure in response to external acoustic pressure, ultimately transitioning into microbubbles when the internal pressure falls below the external pressure.^[^ ^139^ ^]^ During this ADV process, the ultrasonic waves induce a sequence of dynamic actions within the droplets, including oscillation, expansion, contraction, and even rupture of tiny bubbles within the liquid. These events lead to specific chemical reactions and biological effects, with a crucial consequence being the disruption of cellular ultrastructure, ultimately inducing apoptosis and potentially enhancing thrombus dissolution.^[^ ^83^, ^140^ ^]^

**Figure 9:**
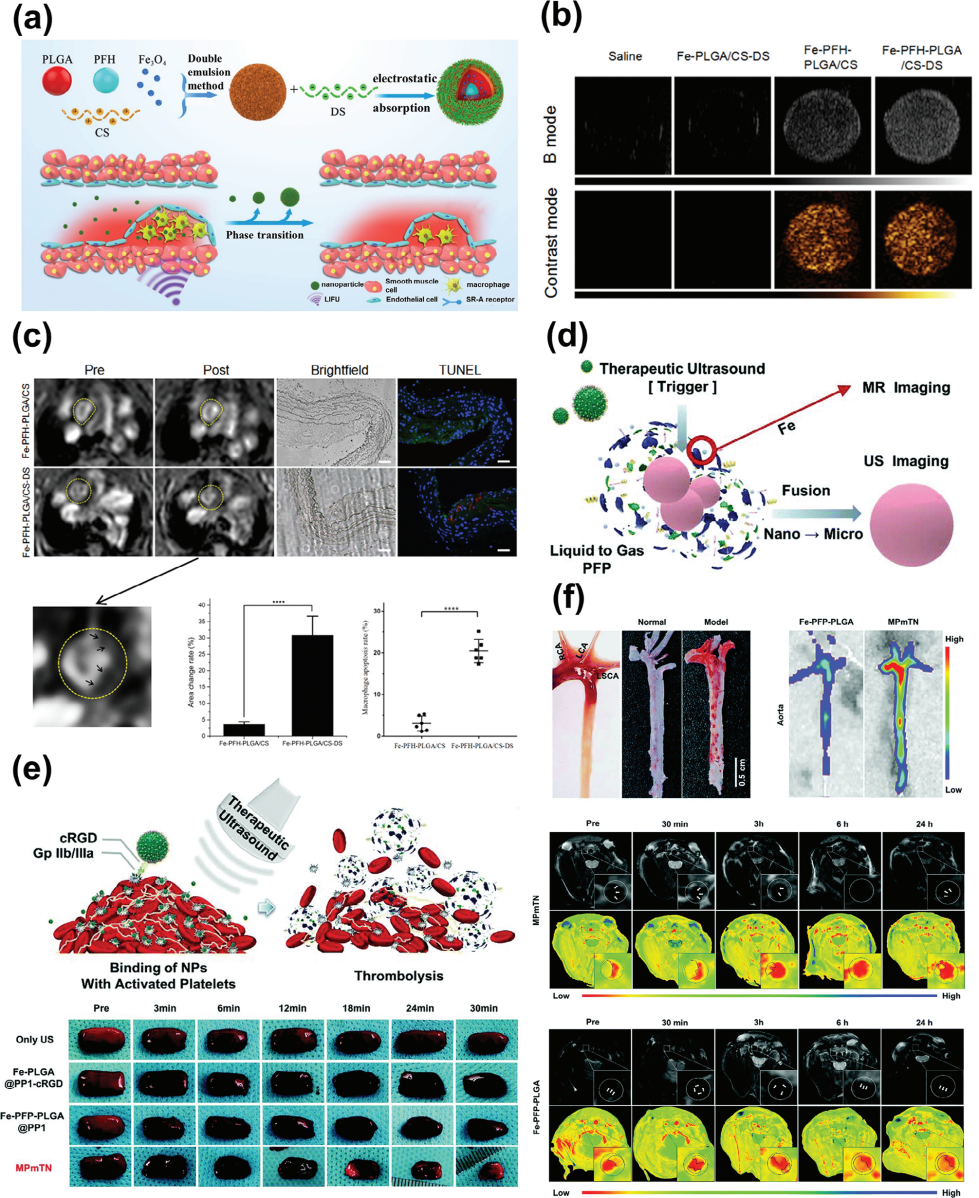
a) Schematic illustration of the preparation and ADV effect of Fe‐PFH‐PLGA/CS‐DS NPs. b) US images of the B mode and contrast mode of Fe‐PFH‐PLGA/CS‐DS NPs in vitro. c) Effects of targeting and treatment in vivo shown by MRI. Reproduced with permission.^[^ ^65^ ^]^ Copyright 2019, American Chemical Society. d) Schematic illustration of ultrasound‐triggered phase transition of MPmTN via the US. e) Schematic of the antithrombotic effect by ADV and the thrombolytic effect of MPmTN in vitro. f) Anti‐atherosclerosis effect of MPmTN in vivo. Reproduced with permission.^[^ ^35^ ^]^ Copyright 2021, Royal Society of Chemistry.

Presently, the realization of ADV relies on liquid fluorocarbons, such as perfluorohexane (PFH) and perfluoropentane (PFP). These fluorocarbons can be encapsulated within nanocarriers, facilitating targeted delivery to disease sites for noninvasive treatment when stimulated by ultrasound. Moreover, they serve a dual purpose by offering diagnostic capabilities as ultrasound contrast agents. Ye et al. devised a nanoparticle formulation encapsulating PFH and IONPs within a PLGA matrix, further modified with chitosan (CS) and dextran sulfate (DS). The nanoparticles exhibited an affinity for scavenger receptor type A (SA‐R) on macrophages at atherosclerotic plaques, resulting in their internalization and subsequent induction of apoptosis through the ADV effect during LIFU irradiation (**Figure** **9**a). Furthermore, this multifunctional nanoparticle enabled bimodal imaging via magnetic resonance and ultrasound (Figure 9b). Upon irradiation with low‐intensity focused ultrasound (LIFU) at a power density of 4 W cm^−^^2^, ultrasound signals were successfully detected in both B‐mode and contrast mode. The results also showed prominent in vivo targeting and therapeutic effects (Figure 9c).^[^ ^65^ ^]^ In a similar vein, Hou et al. loaded PFH and IONPs into biocompatible PLGA‐PEG‐PLGA nanoparticles, which were then surface‐modified with DS to achieve targeted delivery. The introduction of DiR further endowed these nanoparticles with near‐infrared fluorescence (NIRF) imaging capabilities, culminating in the development of a theranostic nanoplatform (FPD@CD NPs) tailored for vulnerable plaques. Notably, the study meticulously investigated and observed the effects of LIFU‐induced ADV at varying power intensities on macrophages in depth. The results indicated varying degrees of macrophage damage under different LIFU power intensities (ranging from 0 to 4 W cm^−^^2^), showing that 2.5 W cm^−^^2^ was the optimal power intensity for LIFU treatment. Furthermore, in animal experiments, FPD@CD NPs exhibited substantial in vivo NIRF/MR imaging capabilities and active targeting efficiency. Following 20 days of LIFU treatment at a power of 2.5 W cm^−^^2^, the experimental group demonstrated a significant reduction in plaque area and a remarkable 49.4% decrease in vascular stenosis, underscoring the significant potential of LIFU as a means to address vulnerable plaques and mitigate acute cardiovascular events.^[^ ^83^ ^]^

In addition to its role in targeting macrophages to mitigate plaque rupture, therapeutic ultrasound can also be directed toward activated platelets in erosion‐prone plaques, thus promoting platelet breakdown and contributing to the stabilization of these vulnerable lesions. Gao et al. ingeniously devised a multifunctional pathology‐mapping theranostic nanoplatform based on Fe_3_O_4_ and PFP, aptly named MPmTN. This innovative platform possesses the unique ability to simultaneously target two distinct types of vulnerable plaques: rupture‐prone plaques characterized by chronic inflammation and macrophage infiltration, and erosion‐prone plaques typified by platelet deposition and thrombosis. Importantly, MPmTN exerts corresponding therapeutic effects on both plaque types. Specifically, MPmTN efficiently homes in on rupture‐prone plaques by leveraging the binding specificity of coupled PP1 to SR‐A. Simultaneously, it accumulates within erosion‐prone plaques through the high adhesion of cRGD peptides to the surface receptor GP IIb/IIIa complex, which is abundantly expressed on activated platelets. Furthermore, the ADV effect triggered by therapeutic ultrasound serves a dual purpose: it induces macrophage apoptosis, resulting in an anti‐inflammatory effect, while also exerting an antiplatelet function that disrupts thrombus formation. The thrombolytic effect and anti‐atherosclerosis effect of the dual‐targeting nanotheranostic based on IONPs are convincingly validated by experimental results (Figure 9d–f).^[^ ^35^ ^]^

The studies described above highlight the immense potential of combining IONPs and ultrasound for the theranostic treatment of atherosclerotic plaques, particularly vulnerable plaques. In this approach, ultrasound serves both as the treatment modality and the imaging strategy, while IONPs primarily function as potent magnetic resonance contrast agents. This dual‐functionality of IONPs compensates for the limitations of ultrasound imaging, allowing for more effective guidance of the treatment process.

#### 4.4.2 Sonodynamic Therapy

**Figure 10:**
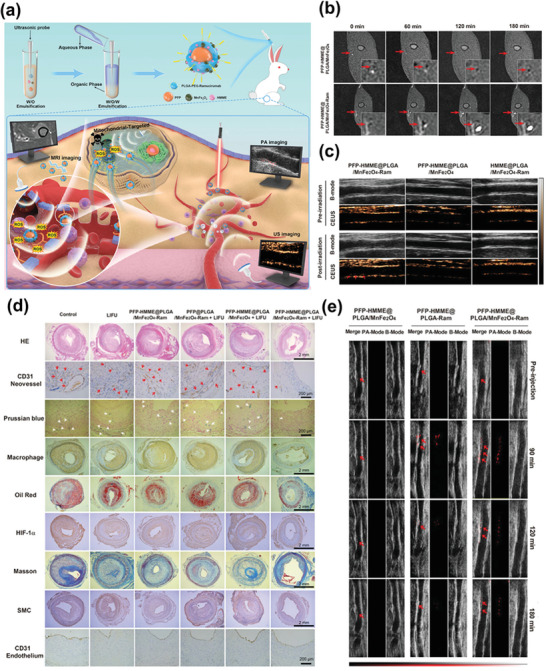
a) Schematic illustration of the synthetic process and corresponding theranostic functionality for PFP–HMME@PLGA/MnFe_2_O_4_–Ram nanoplatform in multi‐effective treatment of atherosclerosis. b) In vivo MRI images of rabbit femoral plaque after injection of different nanoparticles. c) In vivo ultrasound imaging images of rabbit femoral plaque after injection of different nanoparticles. d) Representative histopathological staining of plaque sections presented the treatment outcomes of atherosclerotic rabbits. e) In vivo PA imaging of the rabbit femoral plaques after injection of indicated NPs at different time points. Reproduced under terms of the CC‐BY license.^[^ ^69^ ^]^ Copyright 2021, Published by Wiley‐VCH.

Another compelling strategy for atherosclerosis treatment involving ultrasound is the utilization of nanocarriers loaded with sonosensitizers, enabling sonodynamic therapy (SDT) in the presence of ultrasound. SDT, originating from photodynamic therapy (PDT), leverages sonosensitizers to produce ROS when stimulated by ultrasound, ultimately leading to the destruction of lesional cells. Most of the photosensitizers used in PDT can also serve as sonosensitizers in SDT, and the therapeutic principles of the two modalities are analogous. Yao et al. introduced an innovative strategy for achieving hematoporphyrin monomethyl ether (HMME)‐mediated sonodynamic therapy to combat pathological angiogenesis. The researchers developed a theranostic nanoplatform that encapsulated manganese ferrite (MnFe_2_O_4_), HMME (a representative sonosensitizer), and PFP within PLGA shells. This nanoplatform was further modified with an anti‐VEGFR‐2 antibody, ramucirumab (Ram). This antibody played a dual role—it bound to VEGFR‐2, inactivating VEGF‐mediated downstream signaling pathways to inhibit neovascularization, while also conferring active targeting capabilities to the nanoparticles, allowing them to specifically target aortic endothelial cells. When these theranostic nanoagents were injected into atherosclerotic rabbits and exposed to low‐intensity focused ultrasound (LIFU) irradiation, multimodal imaging involving MRI, PA, and US modalities could be performed. This real‐time imaging provided invaluable guidance for plaque treatment. MRI, with its exceptional soft‐tissue resolution and 3D structural imaging capabilities, was complemented by PAI, an emerging technique. PAI, enhanced by HMME and manganese ferrite, effectively distinguished between neovessels and mature microvasculature. Representative histopathological staining of plaque sections after 28 days of SDT treatment in atherosclerotic rabbits demonstrated the substantial benefits of this targeted nanoparticle‐mediated SDT approach. It significantly suppressed intraplaque hemorrhage and inflammation, inhibited plaque neovascularization, and ultimately enhanced plaque stability. This work showcases promising results of SDT in combating plaque inflammation and neovascularization, offering an excellent example of the synergistic effects achievable through the combination of physical stimulation therapy and pharmacotherapy (**Figure** **10**).^[^ ^69^ ^]^

## 5 Iron Oxide Nanoparticle‐Based Theranostic Nanomedicine through Biologic Therapy

Biologic therapy encompasses a wide range of approaches involving biological macromolecules, such as proteins, nucleic acids, antibodies, as well as living cells and their derivatives, and even biomimetic nanostructures, for the treatment of various diseases. This field has gained immense popularity and holds significant promise as a versatile therapeutic technique with broad applications. What sets biological therapies apart and makes them particularly appealing in clinical practice is their tendency to exhibit lower toxicity and a greater potential for achieving complete disease remission compared to conventional physical or chemical approaches. In this section, we provide an overview of IONP‐based theranostic nanomedicine employed in atherosclerosis treatment through biological therapy. This primarily encompasses HDL mimics, gene therapies, cytokine‐mediated therapy, and various other biological treatment strategies.

### 5.1 HDL‐Mimics: Atherosclerosis Theranostics with HDL‐Inspired Nanoparticles

In recent years, there has been a growing interest in the use of high‐density lipoprotein (HDL)‐mimicking nanoparticles as a promising strategy for diagnosing and treating atherosclerosis. Natural HDL, composed primarily of a core containing cholesteryl esters and triglycerides, enveloped by phospholipids (PL) and apolipoprotein A1 (apoA1), is renowned as a “vascular scavenger.”^[^ ^141^ ^]^ HDL has been found to prevent endothelial dysfunction and facilitate reverse cholesterol transport (RCT), thereby inhibiting the formation of foam cells and ultimately delivering remarkable anti‐atherosclerotic effects.^[^ ^142^ ^]^

Due to the remarkable anti‐atherosclerotic properties exhibited by natural HDL, there has been continuous progress in the development of HDL‐mimicking nanoparticles (NPs). These drug‐free HDL mimics possess biocompatibility, prolonged circulation, and biodegradability, offering inherent therapeutic and targeting capabilities for atherosclerosis. Furthermore, they can serve as carriers for loading diagnostic agents such as Gd, Au, quantum dots, and IONPs, enhancing their imaging capabilities.^[^ ^143^ ^]^ For instance, in 2008, Mulder et al. introduced hydrophobic ligand‐capped Au nanoparticles, FeO nanoparticles, and quantum dots to replace the hydrophobic core of HDL. This innovation allowed for the creation of HDL‐mimicking nanoparticles with unique multimodal imaging abilities, including CT, MRI, or fluorescence imaging.^[^ ^144^ ^]^ HDL‐mimicking NPs loaded with IONPs have also been explored as theranostic agents for atherosclerosis, offering both imaging guidance and targeted therapy. Nandwana et al. developed high‐density lipoprotein‐like magnetic nanostructures (HDL‐MNS), comprising a diagnostic core (Fe_3_O_4_ nanoparticles) and a shell composed of phospholipids and apoA1, mimicking the surface composition of natural HDL. In terms of imaging, HDL‐MNS enabled non‐invasive MRI detection and early diagnosis of macrophage‐rich and cholesterol‐rich atherosclerotic lesions, providing higher contrast than commercial T_2_ contrast agents like Ferumoxytol. In therapeutic applications, HDL‐MNS were capable of internalizing within macrophages, binding to cholesterol, and inducing cholesterol efflux, similar to the action of natural HDL. Importantly, these nanostructures demonstrated biocompatibility. While this study showcased the potential application of HDL‐MNS in atherosclerosis theranostics, further confirmation through in vivo experiments is warranted.^[^ ^84^ ^]^

**Figure 11:**
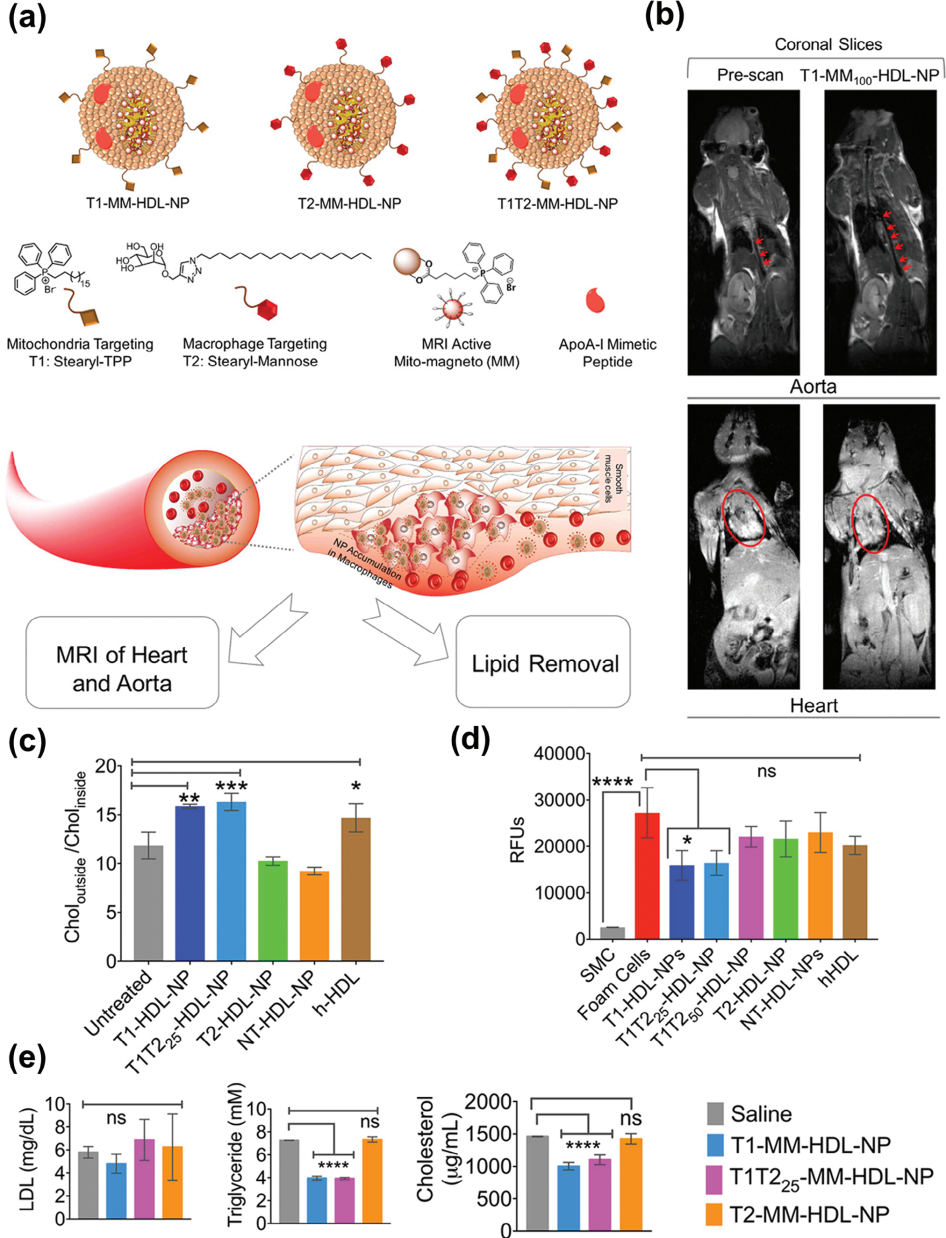
a) Schematic representation of different components and actions of HDL‐mimicking NPs. b) MRI images of coronal slices from BALB/c mice injected with T1‐MM100‐HDL‐NPs. c) In vitro comparison of the cholesterol efflux property of indicated groups. d) In vitro comparison of the antioxidant properties of indicated groups. e) Lipid reduction profiles of BALB/c mice after being treated with MM‐loaded nanostructures for 24 h. Reproduced with permission.^[^ ^61^ ^]^ Copyright 2020, American Chemical Society.

In a subsequent study by Banik et al., a magnetic dual‐targeted HDL‐mimicking NP was designed and evaluated through in vivo imaging and therapeutic efficacy studies in animal models. These HDL‐mimicking NPs featured an IONP core and surface modifications with mannose and triphenylphosphonium bromide. This design enabled them to target plaques through specific binding to mannose receptors on the surface of M2 macrophages and internalize within these macrophages. Additionally, the NPs targeted mitochondria in macrophages via triphenylphosphine, as illustrated in **Figure** **11**a. MRI results revealed the deposition of nanoparticles mainly in the heart and aorta upon injection into mice, leading to enhanced MRI contrast and highlighting their targeting and diagnostic potential (Figure 11b). Moreover, the HDL‐mimicking NPs demonstrated pro‐cholesterol efflux functionality in both in vitro and in vivo experiments, underscoring the importance of mitochondrial targeting for effective lipid removal in vivo (Figure 11c–e).^[^ ^61^ ^]^

The combined approach of using HDL‐mimicking nanoparticles (NPs) along with IONPs demonstrates remarkable in vivo biocompatibility, holding great promise for potential clinical translation. To further enhance the overall therapeutic efficacy of these nano‐formulations, it is possible to load anti‐atherosclerotic drugs into the HDL‐mimicking NPs. Previous studies have explored the delivery of pitavastatin using HDL‐mimicking NPs for targeted atherosclerosis treatment, showcasing a dual effect of inhibiting cholesterol deposition and enhancing exocytosis for effective plaque regression.^[^ ^145^ ^]^ However, these studies did not address the incorporation of IONPs for diagnostic purposes. Therefore, the utilization of HDL‐mimicking NPs for the simultaneous delivery of drugs and imaging agents, creating a safe and efficacy‐enhanced theranostic nanoplatform, remains a promising avenue in atherosclerosis research. This approach warrants further attention and investment to fully realize its potential in the field of atherosclerosis theranostics.

### 5.2 Harnessing Gene Therapy for Atherosclerosis Treatment

Gene therapy, a prospective technique, has been the subject of numerous preclinical studies and clinical trials due to its broad applicability across various diseases.^[^ ^146^ ^]^ Compared to the limited and non‐specific effects of current lipid‐regulating medications, gene therapy can offer a specific and enduring treatment approach for atherosclerosis, allowing the regulation of specific genes in particular cells and the modification of pathological conditions associated with these genes.^[^ ^147^ ^]^ This section focuses on gene therapy combined with IONPs in the field of atherosclerosis theranostics.

#### 5.2.1 Gene Therapy Mediated by Viral Vectors

**Figure 12:**
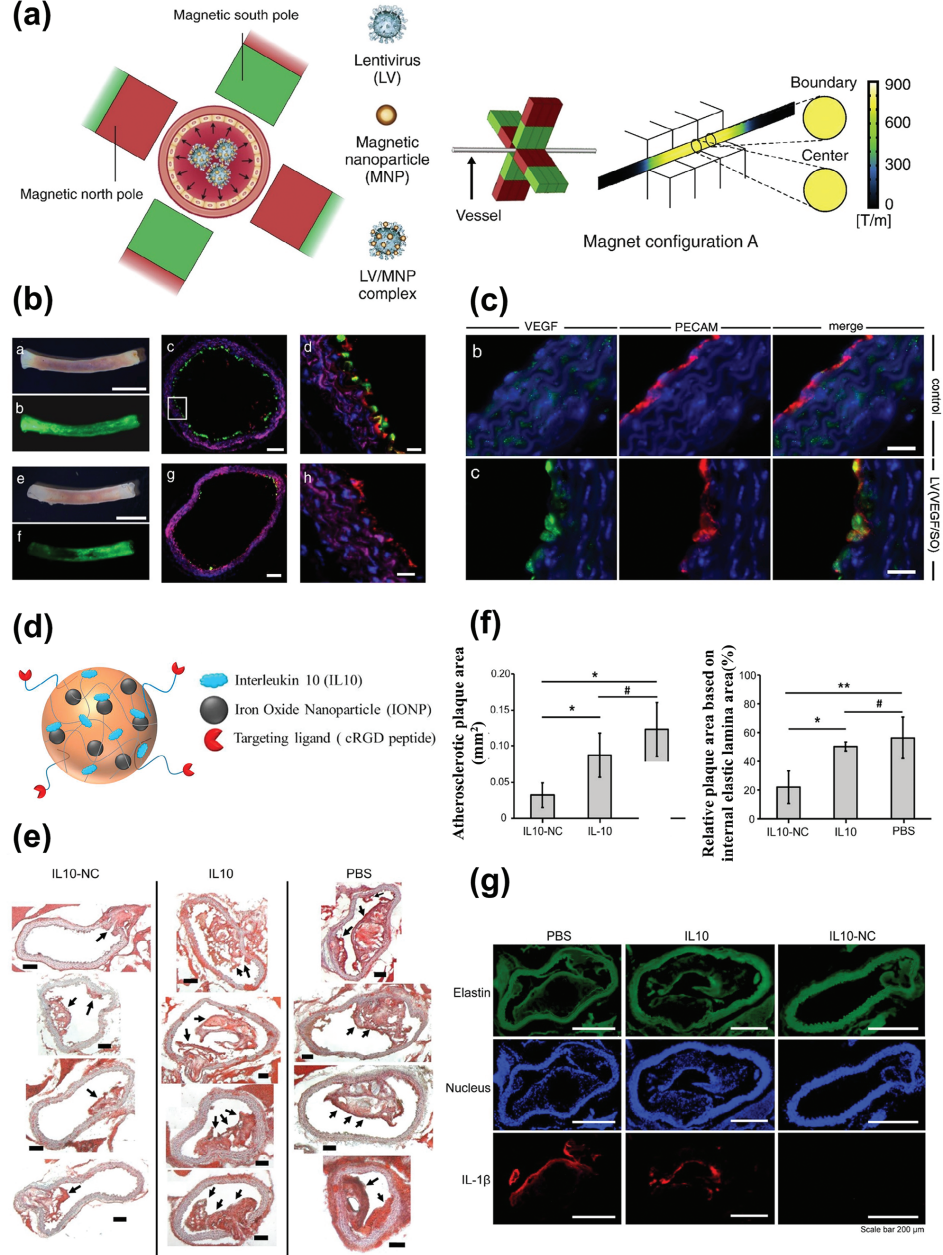
a) Schematic diagram of the radially symmetric transduction of LV/MNP complexes. b) Comparison between magnet configuration A (three images above) and magnet configuration B (three images below) in circumferential lentiviral transduction. c) Immunofluorescence staining of aortic comparing overexpression of VEGF in indicated groups. Reproduced with permission.^[^ ^71^ ^]^ Copyright 2016, Elsevier B.V. d) Schematic diagram of the structure of IL10‐NC. e,f) Oil red O (ORO) staining of plaque and quantitative analysis showing the role in promoting plaque regression of IL10‐NC. g) Immunofluorescence staining of pro‐inflammatory cytokine IL‐1β showing the role in promoting inflammation resolution of IL10‐NC. Reproduced with permission.^[^ ^72^ ^]^ Copyright 2020, Elsevier Ltd.

Effective gene delivery is pivotal for safe and efficient gene therapy. It relies on dependable nanocarriers and precise targeting. Viral vectors, a well‐established technology, have been conventionally used for efficient gene transfection into cells.^[^ ^146a^ ^]^ In a novel approach, Vosen et al. introduced magnetic lentiviral vector complexes (LV/MNPs) for gene therapy, as illustrated in **Figure** **12**a. They initially compared two distinct magnet configurations for lentiviral transduction, selecting the more efficient magnet configuration A for subsequent experiments (Figure 12b). By harnessing the interaction between core‐shell IONPs and a magnetic field, LV/MNPs were propelled along blood vessels and lodged at specific endothelial sites. Consequently, under blood flow conditions, LV/MNPs were transduced into endothelial cells, stimulating the overexpression of eNOS and VEGF in these cells. This led to the restoration of endothelial function and enhancement of vascular health, ultimately contributing to the prevention and alleviation of atherosclerosis (Figure 12b,c).^[^ ^71^ ^]^ Furthermore, the use of IONPs may provide imaging and tracking capabilities, further enhancing targeting and treatment precision.

#### 5.2.2 Gene Therapy Mediated by siRNA

RNA interference (RNAi)‐based gene silencing therapy has gained prominence, especially following the FDA's approval of the siRNA drug (patisiran) in 2018.^[^ ^148^ ^]^ In a study focused on small interfering ribonucleic acid (siRNA) delivery to macrophages, Jia et al. introduced a novel approach utilizing polyethyleneimine (PEI)‐coated superparamagnetic iron oxide nanoparticles (PEI‐SPIONs) as nanocarriers for siRNA delivery. This system aimed to safeguard the integrity and stability of siRNA during delivery while enabling visualization and imaging guidance. PEI, a cationic polymer, forms nanoparticles by electrostatically binding to the strongly negatively charged siRNA, thereby preventing siRNA degradation in vivo. The efficiency of PEI‐SPIONs in mediating siRNA transfection was demonstrated in both RAW264.7 cells and primary macrophages. The inherent phagocytosis of SPIONs by macrophages contributed to effective siRNA transfection by PEI‐SPIONs, resulting in high transfection efficiency. Additionally, the nanocarrier possessed MRI capabilities due to the presence of SPIONs, offering diagnostic insights.^[^ ^85^ ^]^ These findings suggest that PEI‐SPIONs represent a straightforward and efficient siRNA delivery platform for the theranostic treatment of atherosclerosis, with a specific focus on targeting macrophages. While several nanocarriers for siRNA delivery have been reported, such as lipid nanoparticles,^[^ ^149^ ^]^ exosomes,^[^ ^150^ ^]^ chitosan nanoparticles,^[^ ^151^ ^]^ cyclodextrin nanocomposites,^[^ ^152^ ^]^ HDL‐mimicking nanoparticles,^[^ ^145^ ^]^ poly (β‐amino ester) nanoparticles,^[^ ^153^ ^]^ hyaluronan‐based nanosystems,^[^ ^154^ ^]^ and cerium oxide nanowires,^[^ ^155^ ^]^ the majority concentrate solely on atherosclerosis therapy without integrating suitable imaging strategies. This oversight hinders the real‐time monitoring of drug delivery in vivo and the assessment of disease progression. Real‐time monitoring, facilitated by imaging capabilities, can aid in tailoring drug dosages for individual patients and enabling flexible dosage adjustments to enhance therapeutic efficacy while reducing the potential for toxicity.^[^ ^156^ ^]^ In the context of atherosclerosis, it is imperative and promising to explore the amalgamation of effective nanocarriers, along with IONPs known for their superior MRI capabilities, for the co‐delivery of siRNAs, fostering further research in the realm of theranostics.

### 5.3 Cytokine Therapy: Harnessing the Power of Anti‐Inflammatory Biologics

Cytokines hold an essential role in the inflammatory response associated with atherosclerosis, making cytokine‐mediated therapy a compelling avenue for anti‐inflammatory biological intervention.^[^ ^157^ ^]^ Anti‐inflammatory cytokines, such as IL‐10, have been harnessed to exert an atheroprotective effect through the nanotheranostic based on IONPs. Interleukin‐10 (IL‐10), a versatile anti‐inflammatory cytokine, inhibits the production of inflammatory mediators by activated immune cells, thus mitigating inflammation within atherosclerotic lesions.^[^ ^158^ ^]^ Kim et al. encapsulated IL‐10 as a therapeutic agent alongside IONPs as an imaging agent within cRGD peptide‐coupled pluronic‐based nanocarriers (NCs). These NCs were directed to atherosclerotic plaque sites through the binding of cRGD peptides to αvβ3 integrin, which is overexpressed in intraplaque neovascularization. Subsequently, IL‐10 was released to exert its anti‐inflammatory effects. In vitro experiments revealed a release profile characterized by an initial burst followed by sustained release, with no impairment in the ROS scavenging capabilities of IL‐10 due to the presence of NCs. Furthermore, nanocarrier encapsulation significantly improved the pharmacokinetic properties of IL‐10, resulting in an extended serum half‐life for the cytokine. In terms of in vivo therapeutic effects, IL10‐NC treatment substantially reduced aortic root plaque area in mice and decreased levels of IL‐1β, a representative proinflammatory cytokine within plaques, thereby promoting the resolution of inflammation (Figure 12d–g).^[^ ^72^ ^]^ However, it is important to note that this study did not assess the in vivo imaging effects of IONPs.

Numerous strategies have been devised for visualizing inflammation, enabling more precise and controlled anti‐inflammatory treatments for atherosclerosis. IONPs can be ingeniously coupled with various polymers to create ROS‐responsive activatable contrast agents or combined with different contrast agents for multimodal imaging.^[^ ^159^ ^]^ These approaches contribute to the realization of precise image‐guided cytokine‐based anti‐atherosclerotic therapy. In addition, targeting prominent pro‐inflammatory cytokines like IL‐1β, IL‐6, and TNF‐α, monoclonal antibodies with anti‐inflammatory properties, such as tocilizumab,^[^ ^160^ ^]^ infliximab,^[^ ^161^ ^]^ and adalimumab,^[^ ^162^ ^]^ have also shown promise in reducing the incidence of cardiovascular events. Their application with IONPs is also worth exploring in atherosclerosis.

### 5.4 Other Therapeutic Strategies

Among the diverse biologic therapies investigated to enhance cardiovascular health, cell transplantation has emerged as a prominent avenue, with particular emphasis on the pivotal roles of adventitial cell proliferation and migration in vascular endothelium repair.^[^ ^163^ ^]^ Wei et al. leveraged SPIONs to label endothelial progenitor cells, which were subsequently transplanted into a rabbit model of atherosclerosis. This innovative approach aimed to promote vascular repair and regeneration, guided by real‐time MRI. In comparison to conventional endothelial progenitor cell transplantation, this method offers the potential for noninvasive dynamic monitoring of the treatment process. It enables the optimization of the cell transplantation window and provides valuable feedback, positioning it as a promising theranostic strategy for atherosclerosis.^[^ ^86^ ^]^

In another innovative study, Cohen's group harnessed the anti‐inflammatory properties of apoptotic cells to develop liposomes containing iron oxide nanoparticles. These liposome nanoparticles, by presenting phosphatidylserine on their surface, mimicked apoptotic cells and transmitted death signals to macrophages. Upon recognition and phagocytosis by macrophages, these liposomes effectively inhibited the release of pro‐inflammatory cytokines while promoting the secretion of anti‐inflammatory cytokines. Additionally, these liposomes exhibited pro‐angiogenic effects in a rat model of acute myocardial infarction and were trackable via in vivo MRI.^[^ ^87^ ^]^ This multifaceted approach holds promise as it combines anti‐inflammatory and pro‐angiogenic effects while enabling noninvasive imaging for therapeutic guidance.

## 6 Challenges and Prospects: Navigating the Path Ahead

In this comprehensive review, we have embarked on an in‐depth exploration of the recent strides made in theranostic nanoplatforms centered around iron oxide nanoparticles (IONPs) for the treatment of atherosclerosis. Our examination has been meticulously framed within the context of three distinct therapeutic strategies: chemical therapy, physical stimulation therapy, and biological therapy. Over the past decade, IONPs have emerged as a compelling instrument, not only for the diagnosis but also for the treatment of atherosclerosis. Their allure in this context is underpinned by the escalating fascination with magnetic nanoparticles in the realm of disease diagnosis and therapeutic intervention. Additionally, the intrinsic attributes of magnetic nanoparticles, such as their inherent propensity to congregate at lesion sites and their ready uptake by macrophages, seamlessly align with the intricate pathophysiology of atherosclerosis.

Within the panorama of theranostic nanomedicines that we have scrutinized, IONPs assume the pivotal role of exceptional magnetic resonance contrast agents. This role is nothing short of indispensable for the real‐time tracking of nanomedicine distribution and therapeutic efficacy. It furnishes a vital feedback mechanism that empowers the fine‐tuning of treatment doses and schedules, ushering in the era of personalized medicine. Beyond their role as mere imaging agents, IONPs contribute an additional layer of versatility as reliable nanocarriers. They stand as stalwart transporters for an extensive array of therapeutic agents, encompassing clinical drugs, natural bioactive molecules, nanozymes, photosensitizers, phase‐change materials, nucleic acids, and cytokines. Their manifold advantages encompass impeccable biocompatibility, facile surface modification, a rich assortment of synthesis methodologies, and the capacity for scalable production. Profiting from their magnetic targeting prowess, IONPs guarantee the efficient conveyance of therapeutic payloads to the site of affliction, thereby amplifying the therapeutic endeavor. The allure of IONPs doesn't stop at their role as carriers or imaging agents; their photothermal and magnetothermal properties cast them as potential therapeutic agents in photothermal therapy (PTT) and magnetothermal therapy (MHT), opening new horizons for multifaceted interventions in atherosclerosis.

**Figure 13:**
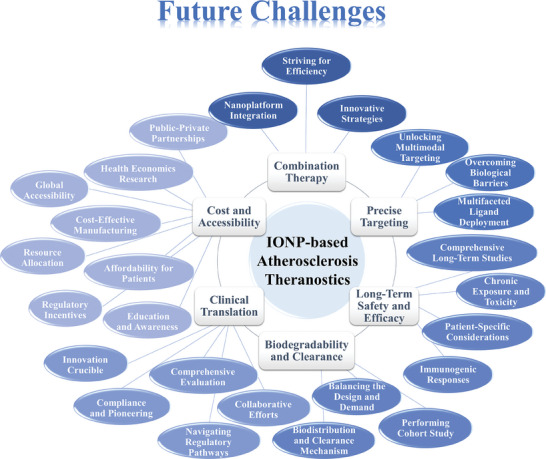
The future challenges and uncharted territories for the development of IONP‐based Atherosclerosis Theranostics.

As we stand on the precipice of an exciting era in atherosclerosis theranostics, it is imperative to acknowledge the challenges that await and to chart a course for the future. While the potential of iron oxide‐based nanoparticles in diagnosing and treating atherosclerosis is undeniable, the journey ahead is not without hurdles. Here, in our quest for innovation and improvement, we must acknowledge the challenges and uncharted territories that lie ahead (**Figure** **13**).

### 6.1 Combination Therapy

Combination therapy stands as a beacon of potential in the realm of therapeutic interventions, recognized for its capacity to elevate treatment outcomes. However, the landscape of IONP‐based theranostic nanomedicines has witnessed a relatively measured exploration of this avenue. This nuanced engagement with combination therapy can be attributed to several factors that merit consideration:

#### 6.1.1 Intricacies of Nanoplatform Integration

At the heart of the challenge lies the intricate task of seamlessly integrating therapeutic and diagnostic functionalities within nanoplatforms. This intricate fusion has the potential to introduce complexities into the synthesis, characterization, and pharmacokinetics of nanomedicines. The multifaceted nature of these systems necessitates meticulous attention to detail.

#### 6.1.2 Striving for Efficiency

Effective combination therapies should be characterized by maximal therapeutic efficiency achieved through minimal components. This efficiency‐driven approach underscores the importance of leveraging multifunctional molecules that can fulfill multiple roles within the nanoplatform. Such molecules can streamline the complexity of nanosystems while preserving their therapeutic efficacy.

#### 6.1.3 Innovative Strategies

To navigate the complexities and intricacies inherent in the convergence of therapeutic and diagnostic functions, innovative strategies are imperative. These strategies should seek to strike a harmonious balance between the multifaceted nature of nanosystems and the imperative of achieving therapeutic efficacy. The development of novel approaches and multifunctional molecules is essential in this pursuit.

### 6.2 Precise Targeting

Achieving precision targeting is paramount in the endeavor to concentrate nanomedicines precisely at the hallowed grounds of atherosclerotic plaque sites. While single‐ligand targeting has showcased promise within the controlled confines of in vitro experimentation, its translation to in vivo applicability confronts formidable biological barriers that demand innovative solutions. Here, we delve into the complexities of precision targeting and the strategies that offer a glimpse into its attainment:

#### 6.2.1 Overcoming Biological Barriers

The formidable biological barriers that guard atherosclerotic plaque sites pose a significant challenge to the success of targeting strategies. To overcome these hurdles, one promising approach is the fusion of a cell membrane coating with a targeting ligand. This ingenious stratagem capitalizes on the cell's natural camouflage, allowing the nanomedicine to navigate biological defenses with greater finesse.

#### 6.2.2 Multifaceted Ligand Deployment

Another avenue to enhance precision targeting involves the strategic deployment of multiple ligands on the surfaces of nanomedicines. This multifaceted approach equips nanomedicines with an arsenal of targeting mechanisms, increasing their chances of breaching the biological barriers. By orchestrating the concerted efforts of diverse ligands, nanomedicine can augment its accumulation at plaque sites, enhancing its therapeutic impact.

#### 6.2.3 Unlocking Multimodal Targeting

Precision diagnosis of atherosclerotic plaques at various stages emerges as a promising vista. This can be achieved by orchestrating the targeting of diverse markers or multiple targets on the same cellular canvas. Multimodal targeting strategies enable the nanomedicine to discern the nuanced characteristics of plaques, facilitating their precise diagnosis across different stages of atherosclerosis.

### 6.3 Long‐Term Safety and Efficacy

The pursuit of IONP‐based theranostic nanomedicines is underpinned by a commitment to patient safety and long‐term efficacy. As these innovative nanosystems progress toward clinical application, several considerations and challenges must be addressed:

#### 6.3.1 Comprehensive Long‐Term Studies

Rigorous and comprehensive long‐term studies are indispensable to assess the safety and efficacy of IONP‐based theranostic nanomedicines. These studies should span extended periods, tracking the fate of nanoparticles within the body. It is essential to monitor potential adverse effects, evaluate nanoparticle clearance pathways, and gain insights into their long‐term behavior in various tissues.

#### 6.3.2 Chronic Exposure and Toxicity

Persistent exposure to nanoparticles raises legitimate concerns necessitating thorough examination. Although short‐term studies have showcased the commendable biocompatibility of IONPs, their long‐term behavior and effects may deviate. It is imperative to conduct comprehensive assessments to gauge the potential accumulation of nanoparticles within organs or tissues and to ascertain any related toxicities, as these factors are pivotal in ensuring long‐term safety.

#### 6.3.3 Immunogenic Responses

Comprehending the immunogenic responses triggered by IONP‐based nanomedicines is of paramount importance. The immune system's reaction to nanoparticles can evolve, possibly giving rise to unforeseen responses or sensitization. Therefore, a thorough exploration of immunogenicity dynamics and the formulation of strategies to mitigate adverse immune reactions are essential.

#### 6.3.4 Patient‐Specific Considerations

Acknowledging the potential variability in patient responses to IONP‐based theranostic nanomedicines is pivotal. Customizing treatment plans and monitoring strategies to accommodate these individual differences forms an integral component of the long‐term safety and efficacy framework. The sustained safety and effectiveness of these nanosystems are likely to require an iterative refinement process. As data accumulates and insights surface from clinical applications, nanoplatforms can be fine‐tuned, and safety profiles can be further improved.

### 6.4 Biodegradability and Clearance

The mechanisms of excretion and the biodegradability for nanoparticles are highly concerned by regulatory bodies and could significantly impact the preclinical and clinical development of nanotheranostics. Especially for IONPs, the potential side effects of themselves and their biodegradation products make evaluating their biodegradability and understanding their clearance pathways in vivo an indispensable aspect and a central challenge. This section briefly discusses the following aspects:

#### 6.4.1 Biodistribution and Clearance Mechanism of Iron Oxide‐Based Nanoparticles

According to previous research, most IONPs administered systemically are mainly absorbed by the mononuclear phagocytic system (MPS), and accumulate in the liver and spleen. IONPs are taken up into Kupffer cells of the liver sinusoid and macrophages of the splenic red pulp through endocytosis.^[^ ^164^ ^]^ Their biodegradation mechanism is thought to be analogous to the metabolism of ferritin. After being internalized by cells, IONPs are digested by lysosomal enzymes to release iron ions. Subsequently, excess iron ions are regulated by various clearance mechanisms in vivo, stored or utilized.^[^ ^165^ ^]^ Furthermore, research has also shown that when the iron content of the body exceeds the available apoferritin, it tends to form small insoluble aggregates in liver cells and slowly release iron ions.^[^ ^166^ ^]^ Additionally, another metabolic pathway is direct and rapid excretion through the kidneys, but this usually requires the particle size of nanoparticles to be below 10 nm.

#### 6.4.2 Balancing the Design and Demand

Many factors can affect the biological distribution and degradation of IONPs or IONPs‐based nanotheranostics, including size and shape, surface charge, coating molecules, targeting and administration approaches, etc.^[^ ^167^ ^]^ These factors are carefully considered to design and optimize the biodistribution and metabolic pathways of nanoparticles. Notably, although rapid elimination is a safer and simpler strategy, many nanomedicines are designed to obtain a longer circulation time, accumulate in diseased tissues, and gradually degrade, which is also reflected in many converted clinical drugs.^[^ ^168^ ^]^ This indicates that nanotheranostics developed for clinical conversion need to optimize degradation and clearance rates to match retention time with the actual demand.

#### 6.4.3 Performing the Cohort Study

With the development of tools such as data mining and deep learning, studying and analyzing a large amount of research data on the biodegradation and clearance of IONPs to obtain predictive results on the biological metabolic pathways of designed nanoparticles may be a potential method. For example, based on the three dimensions of IONPs properties (such as size, shape, surface characteristics, etc.), disease type and stage, and animal species, existing results are stratified, and a model that can predict the metabolic parameters of IONPs is established through continuous optimization. For clinical applications, this method contributes to optimizing the design of nanoparticles and selecting appropriate administration frequencies and doses, to minimize the potential harm caused by IONPs and their biodegradation products. Achieving this idea is established on the data of a large number of experimental cohort studies, so at this stage, more experiments on the in vivo degradation and clearance of IONPs are worth promoting and encouraging.

### 6.5 Clinical Translation and Regulatory Challenges

The translation of theranostic nanosystems from promising preclinical results to clinical reality presents multifaceted challenges and requires a concerted effort from various stakeholders. Here, we delve into the intricacies of clinical translation and the regulatory landscape, acknowledging the hurdles and opportunities they encompass:

#### 6.5.1 Navigating Regulatory Pathways

The journey from preclinical research to clinical trials and eventual FDA approval is akin to breaching a formidable citadel. Theranostic nanosystems, with their multifaceted nature, introduce complexities that demand careful consideration. Conventional regulatory frameworks must evolve to encompass the convergence of diagnostics and therapeutics within a single nanoplatform. Establishing a nuanced regulatory framework that bridges these domains is paramount.

#### 6.5.2 Comprehensive Evaluation

The inherent complexity of theranostic nanosystems, comprising multiple components with intricate physical and chemical profiles, necessitates meticulous characterization and comprehensive evaluations. These evaluations encompass biocompatibility assessments, potential immunogenic responses, and the specter of adverse effects. Establishing independent and all‐encompassing evaluation criteria for nanoparticle biosafety is an urgent need, especially in the realm of composite nanosystems.

#### 6.5.3 Innovation Crucible

The crucible of innovation beckons in the domains of nanoparticle design, therapeutic efficacy, and biosafety assessments. Continued innovation is indispensable in propelling nanomedicine closer to its tryst with clinical realization. Novel approaches to enhance therapeutic efficacy, improve targeting precision, and mitigate potential risks will be pivotal.

#### 6.5.4 Collaborative Efforts

The successful clinical translation of theranostic nanosystems necessitates close collaboration among scientists, clinicians, regulatory authorities, and policymakers. Interdisciplinary cooperation can align their efforts to create a streamlined approval process. Ongoing dialogue and information sharing can help in crafting flexible regulatory strategies that accommodate the distinct characteristics of theranostic nanosystems.

#### 6.5.5 Compliance and Pioneering

Straddling the boundary of diagnostics and therapeutics necessitates both compliance with existing regulations and the pioneering of new ones tailored to the distinctive characteristics of these nanosystems. Ensuring adherence to established regulatory guidelines while actively participating in the creation of novel frameworks is a formidable but essential task.

### 6.6 Cost and Accessibility

The considerations of cost and accessibility are central factors in determining the real‐world impact and adoption of theranostic nanosystems. To ensure that these advanced treatments contribute meaningfully to healthcare, several key aspects must be addressed:

#### 6.6.1 Cost‐Effective Manufacturing

Developing cost‐effective manufacturing processes for theranostic nanosystems is imperative. Innovative techniques, economies of scale, and efficient production methods should be explored to reduce production costs. Collaboration between academia, industry, and regulatory bodies can facilitate the development of streamlined and affordable manufacturing processes.

#### 6.6.2 Affordability for Patients

Making theranostic nanosystems financially accessible to patients is essential. Strategies such as price regulation, insurance coverage, and subsidies can help alleviate the financial burden on individuals. Additionally, partnerships between pharmaceutical companies and healthcare providers can lead to cost‐sharing models that enhance affordability.

#### 6.6.3 Global Accessibility

Ensuring global accessibility to theranostic nanomedicines is a paramount goal. Addressing disparities in access between developed and developing regions is essential. International collaborations, technology transfer initiatives, and partnerships with organizations dedicated to global health equity can help bridge these gaps.

#### 6.6.4 Resource Allocation

Healthcare systems and policymakers should allocate resources strategically to integrate theranostic nanomedicines into standard care pathways. Investments in research, infrastructure, and workforce training are necessary to build the foundation for their effective utilization.

#### 6.6.5 Health Economics Research

Conducting health economics research is critical to evaluating the cost‐effectiveness of theranostic nanosystems. Analyzing their long‐term benefits, including reduced hospitalization and improved patient outcomes, can provide evidence to support their inclusion in healthcare budgets.

#### 6.6.6 Regulatory Incentives

Regulatory agencies can play a role in incentivizing the development and affordability of theranostic nanosystems. Expedited approval processes, fast‐track designations, and orphan drug status for specific applications can encourage innovation and cost‐conscious development.

#### 6.6.7 Public‐Private Partnerships

Collaborations between public and private sectors can drive affordability and accessibility. Joint initiatives that combine government funding, industry expertise, and academic research can accelerate the development and deployment of theranostic nanomedicines.

#### 6.6.8 Education and Awareness

Educating healthcare providers, policymakers, and the public about the benefits and cost‐effectiveness of theranostic nanosystems is essential. Robust communication and awareness campaigns can help garner support and foster understanding.

In conclusion, theranostic nanoplatforms anchored in IONPs hold immense promise in the realm of atherosclerosis diagnosis and treatment. They are the vanguard of personalized medicine, offering the potential for enhanced therapeutic outcomes and real‐time monitoring of disease progression. As we continue to explore and refine these nanosystems, addressing the challenges and limitations we have illuminated will be the crucible in which their full potential is unlocked. These challenges, formidable though they may be, are stepping stones on our path to harnessing the transformative power of theranostic nanosystems. We are poised at the threshold of a new era in atherosclerosis theranostics, where science, innovation, and clinical application converge to redefine patient care and well‐being.

## Conflict of Interest

The authors declare no conflict of interest.
